# The evolution of cooperation in the unidirectional division of labour on a tree network

**DOI:** 10.1098/rsos.230830

**Published:** 2023-11-22

**Authors:** Md Sams Afif Nirjhor, Mayuko Nakamaru

**Affiliations:** School of Environment and Society, Tokyo Institute of Technology, 3-3-6, Shibaura, Minato, Tokyo 108-0023, Japan

**Keywords:** cooperation in tree graph network, replicator equation of asymmetric games, sanction systems, supply chain

## Abstract

Division of labour on complex networks is rarely investigated using evolutionary game theory. We investigate a division of labour where divided roles are assigned to groups on the nodes of a general unidirectional finite tree graph network. From the network’s original node, a task flows and is divided along the branches. A player is randomly selected in each group of cooperators and defectors, who receives a benefit from a cooperator in the upstream group and a part of the task. A cooperator completes their part by paying a cost and then passing it downstream until the entire task is completed. Defectors do not do anything and the division of labour stops, causing all groups to suffer losses due to the incomplete task. We develop a novel method to analyse the local stability in this general tree. We discover that not the benefits but the costs of the cooperation influence the evolution of cooperation, and defections in groups that are directly related to that group’s task cause damage to players in that group. We introduce two sanction systems, one of which induces the evolution of cooperation more than the system without sanctions, and promote the coexistence of cooperator and defector groups.

## 1. Introduction

According to historical and anthropological material that has been provided in earlier studies regarding our modern human civilization, division of labour was evident in many pre-industrial societies and was connected to their evolution (e.g. [1,2]). The division of labour existed even in civilizations without organized systems like governments [3]. The division of labour results in specialization, which is the foundation of the contemporary, industry-centred civilization. Trade and specialization occur both inside and between industries in the global production networks. Thus, there is a division of labour that is both national and global. Because of the ever-increasing similarities among the modern countries’ socio-economic systems, specialization in and among industries are becoming more frequent [4]. The division of labour is a prerequisite for some social systems. One of the examples is supply chain in the economic system which originates from intraproduct specialization (e.g. [4,5]). The supply chain has several stakeholders and consists of several suppliers, manufacturers, distributors, retailers and customers on the basis [5]. The multi-layer chain subcontracting system, where a client places the work order with a main contractor who in turn places the work order with subcontractors, which continues until the work reaches the bottom-layer contractors, also depends on intraproduct specialization, thus it is also a case of division of labour (e.g. [6]). The bureaucratic system has a hierarchical structure, based on the division of labour, where the specialization in the specific tasks or official duties leads to the success of the bureaucratic system or organization (e.g. [7–9]).

To complete the division of labour, mutual trust and cooperation between groups as well as within groups in the social system is crucial. Even though mutual cooperation is the foundation of human civilization [10] and our society has prospered through mutual cooperation [11], individual rationality, on the contrary, often leads to collective irrationality, and this causes the social dilemma or free riding of defectors. The theoretical and experimental studies on the evolution of cooperation have investigated what mechanisms promote the evolution of cooperation and hinder defectors and free-riders on the basis of the evolutionary game theory.

Nowak [12] and Rand & Nowak [13] describe that there are five mechanisms to encourage cooperation; these are kin selection [14], direct reciprocity (e.g. [15–17]), indirect reciprocity (e.g. [18–22]), network reciprocity (e.g. [23–29]) and group selection (e.g. [30,31]). In addition to these, the utilization of sanctions to enforce cooperation has also been investigated both theoretically and empirically (e.g. [32–44]).

The division of labour exists in our society and cooperation is key there. The division of labour has been studied lately in terms of evolutionary game theory. There are some theoretical works dealing with the division of labour (e.g. [45–52]), which considered the evolution of the division of labour when there are two or three social roles. Basically, many previous studies assumed a non-structural division of labour among an infinite number of players (e.g. [51,53]). The evolutionary dynamics of the division of labour on a cycle network has also been studied using the division of labour game in which two players, who play different roles from each other, get a higher payoff than two players who play the same role, and it is assumed that each player can choose one of two roles and plays the game with their neighbours on the cycle network [52]. The division of labour is a premise of supply chain, which has been investigated especially in management science, and no studies about supply chain with network structure have been carried out using evolutionary game theory yet (e.g. [54]).

In reality, there are often more than two or three social roles on network structures in the division of labour. However, the division of labour with network structure where different roles are represented by different nodes is largely unexplored in terms of evolutionary game theory. The linear division of labour was explored by Nakamaru *et al.* [55] and Nirjhor & Nakamaru [56] employing evolutionary game theory. Nakamaru *et al.* [55] exemplified the Japanese industrial waste treatment system assuming that there are three roles, and Nirjhor & Nakamaru [56] studied the general case in which there are a finite number of roles. Here, each subtask should be finished in order, consecutively. They selected a finite number of subtasks in an attempt to simplify the model and made the assumption that there are players in each group, where one subtask is assigned to the players of each group. A player in the upstream group randomly chooses a player in the immediate downstream group. If the player in the upstream group is a cooperator, the player completes his task by paying a cost of cooperation and passes it to the downstream player who receives the benefit. Then if the upstream player is a defector, the player neither completes any task nor passes it to the downstream player. If all players who are chosen from all groups are cooperators, the division of labour is completed, otherwise, it fails. Since the payoffs depend on their tasks and are asymmetric for members of various groups, this system may be represented using the replicator equations for asymmetric games. Through the use of replicator equations for asymmetric games, they investigated whether the two present sanction systems, the defector sanction system and the premier sanction system can promote the evolution of cooperation. In the premier sanction system, the player in the first group is penalized by the supervisor if defection is discovered in the linear chain. If a defector is discovered under the defector sanction system, they are punished by the supervision. Though both sanction systems are capable of enforcing cooperation more than the system without sanction, when it is practically impossible to monitor and identify defectors the first role sanction system has been proven to be more successful at ensuring the evolution of cooperation than the defector sanction system. Nirjhor & Nakamaru [56] found out that the premier sanction system is incapable of creating cooperation when the cooperators are rare initially, however, the defector sanction system is capable of creating cooperation. Nirjhor & Nakamaru [56] have also found out that the benefit given to a player by a cooperator in the former group has no effect on the evolution of cooperation, and showed that the defector sanction system promotes the coexistence of groups full of cooperators and a group full of defectors when the cost of cooperation increases in the downstream groups.

The study of the evolution of cooperation in the division of labour on the tree graph network has significantly more applicability than the linear network of Nirjhor & Nakamaru [56]. This is because the division of labour on a tree graph is more common than the linear division of labour in our real world. For example, governments in most countries have a system of hierarchy. There is a governmental head or premier, under whom there are several departments, each of which has a head of its own. Each department then breaks down into several sub-departments and until the root level. Therefore, a government system can be considered as a finite tree graph network, which has an origin at the premier and a finite number of branches. Each of the nodes represents a government official. Most governmental action can be considered as a division of labour [58], which is ordered by a head and then passes through the downstream nodes and gets fulfilled at some terminal node. Therefore, for an order to be carried out cooperation is very important. Often a single order is carried down and executed by a single linear chain of command which is similar to the linear division of labour which was our previous study [56]. However, to see a governmental system as a whole, a tree graph structure is suitable.

The minimum structure of the supply chain is linear, consisting of suppliers, manufacturers, distributors, retailers and customers (e.g. [54]). However, network-focused models can depict a better image of supply chain than linear chain models [59]. The supply chain looks more like a tree than a linear pipeline or chain [60,61]. Therefore, a tree network is capable of depicting the linear network of the supply chain, as well as more general cases. When considering a unidirectional tree network as a supply chain, from the perspective of a player in a terminal node, it is a linear network of the supply chain. However, a player situated in some earlier node can divide the goods along the process links [54] as well as the labour or responsibility required to improve upon those according to the need. In addition, the multilayered subcontract can be depicted by a tree network [6].

In this paper, we study the evolution of cooperation in the division of labour on a general tree graph. No previous models capture all the aspects of a general tree graph in the supply chain (e.g. [54]), and our study challenges this problem by means of the evolutionary game theory.

## 2. Baseline model and results

### 2.1. Model assumption

**Figure 1.**
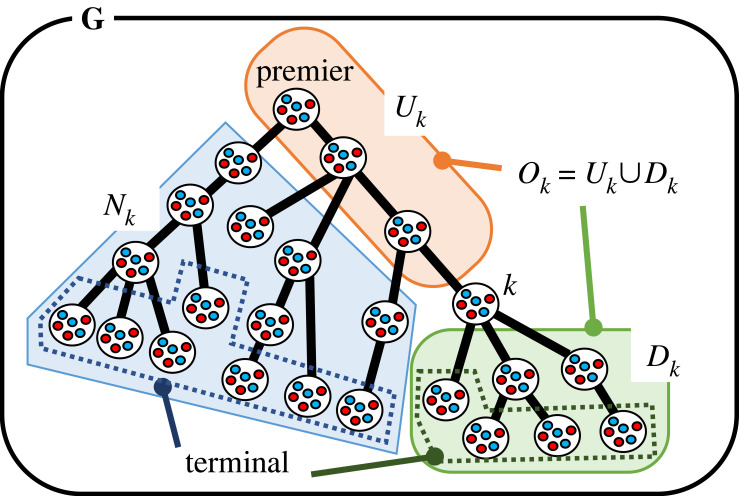
Division of labour in a downstream tree graph. *k* is the focal group, and based on it we divide the tree graph in several sets of nodes for the purpose of generalization. The upstream of *k*, *U*_*k*_ is a linear network from the premier, the downstream of *k*, *D*_*k*_ is a tree graph network. *U*_*k*_ and *D*_*k*_ makes the *O*_*k*_. *N*_*k*_ is created with the groups that are not present in $O_{k}\cup k$.

We take a model where the whole task is divided and assigned to the groups who are present in the nodes of a connected directed tree graph. The model structure is shown in figure 1. In this model, a task is always passed from the upstream to the downstream, never from downstream to upstream, hence, this is unidirectional. There is a unique central node in this graph, and from there branching starts. **G** is the set of nodes in the tree graph (figure 1). Each of the nodes has a group of players, each group consists of cooperators and defectors, and the group population is infinite. *p* is the index of the original node which represents the premier group. In the beginning, the whole task, which can be a service or development of a product, is assigned to the original group, where a player is randomly selected, who gets a benefit *b*_*p*_. The player receives the benefit for receiving the task and it can be considered as the value of the task [56]. For example, in the multi-layered contract development system, the orderer pays the contract money, *b*_*p*_, to the contractor, which pays the money to the subcontractors and it continues when the terminal sub-subcontractors receive the money and do their tasks. In the industrial waste disposal system, it can be considered as the benefit from the product, from which the waste was produced [55].

If the chosen player in a group is a cooperator, they pay a cost of cooperation *x*_*p*_ to improve upon the task. Then they divide the task and pass the task to one or more downstream branches. Each player is chosen randomly from each of the receiving downstream groups, and also receives a benefit, as each has received the task. Table 1 shows the payoff of a cooperator in the premier group when all players chosen from all downstream groups are cooperators: *b*_*p*_ − *x*_*p*_ (table 1).

**Table 1.** The payoff matrix in baseline system for premier.

cases	all being cooperator except the premier	a defector after the premier
premier cooperator	*b*_*p*_ − *x*_*p*_	*b*_*p*_ − *x*_*p*_ − *g*_*op*_
premier defector	*b*_*p*_ − *g*_*p*_	*b*_*p*_ − *g*_*p*_

**Table 2.** Losses to the players in the groups in figure 2.

group *k*	state	*γ*_*k*_	*g*_*ok*_	*g*_*nk*_	relative equilibrium
premier	cooperation	0	$g_{k_{12}}+g_{k_{2}}$	0	${mix}_{D_{\;p}}$
*k*_1_	cooperation	0	$g_{k_{12}}$	$g_{k_{2}}$	${mix}_{D_{k_{1}}}$
*k*_11_	cooperation	0	0	$g_{k_{12}}+g_{k_{2}}$	${allc}_{k_{11}}$
*k*_12_	defection	$g_{k_{12}}$	0	$g_{k_{2}}$	${mix}_{U_{k_{12}}\cup \{k_{12}\}}$
*k*_2_	defection	$g_{k_{2}}$	0	$g_{k_{12}}$	${mix}_{U_{k_{2}}\cup \{k_{2}\}}$
*k*_21_	neutral	$g_{k_{21}}$	0	$g_{k_{12}}$+$g_{k_{22}}$	${mix}_{U_{k_{21}}\cup \{k_{21}\}}$
*k*_22_	neutral	$g_{k_{22}}$	0	$g_{k_{12}}+g_{k_{21}}$	${mix}_{U_{k_{22}}\cup \{k_{22}\}}$

If a defector is chosen from the premier group, the defector does not produce any benefit paying a cost of cooperation, and the division of labour does not start. As a result, the player from the premier group will suffer from the loss, *g*_*p*_, which can be interpreted as the damage or the bad reputation from the incomplete tasks of the whole system (table 1). Then, a player in the downstream group *k* (*k* ≠ *p*) suffers from the loss, *g*_*k*_, and it is assumed that $g_{\;p}=\sum g_{\;j}$, where *j*s are the groups present in the immediate branching of the premier. We will explain the assumption of the loss caused by defection later.

Even though a cooperator is chosen from the premier group, the cooperator suffers from the loss, *g*_*op*_ if a defector is chosen in the downstream groups in *D*_*p*_ and the division of labour stops there (table 1). To define *D*_*p*_, we have to define *O*_*k*_, *U*_*k*_, *D*_*k*_ and *N*_*k*_ with respect to group *k* at first (figure 1). *O*_*k*_ is the set of nodes that create *k*’s connection with the premier and are present on the branches originating from *k* if *k* is not premier (figure 1). *U*_*k*_ is the set of the upstream nodes of *O*_*k*_ with respect to *k* and these nodes are in a linear division of labour with respect to *k*. *D*_*k*_ is the set of the downstream nodes of *O*_*k*_ with respect to *k*. Therefore, *O*_*k*_ = $U_{k}\cup D_{k}$ (figure 1). When a defector is chosen from group *k* during that particular task, *D*_*k*_’s groups become neutral, as they do not have a choice. $N_{k}=G-(O_{k}\cup \{k\})$ is the set of nodes that do not have the direct or indirect interaction with *k*.

If *k* is premier or *p*, both *U*_*p*_ and *N*_*p*_ do not exist, and *D*_*p*_ is equal to *O*_*p*_. If *k* is terminal or *t*, *D*_*t*_ does not exist, *U*_*t*_ is equal to *O*_*t*_, and $N_{t}=G-(U_{t}\cup \{t\})$.

We consider *k* is the index of our focus group, and this focus group can be any group in the graph. The value, *b*_*k*_, is the benefit of the player chosen from the group *k*, given by the cooperator in the nearest upstream group *u* with paying a cost of cooperation *x*_*u*_. The benefit can also be considered as the value of the task. If the player in group *k* is also a cooperator, he pays the cost of cooperation *x*_*k*_ to produce a new task or value, *b*_*kk*_, and then passes it to his group’s branch/branches if group *k* is not terminal. The net benefit of the cooperator in group *k* is *b*_*k*_ − *x*_*k*_ if all other players are cooperators. If group *k* has two nearest downstream groups, for example, and they are named group A and group B, a cooperator in the group *k* gives a benefit to each player in two groups. There are two possible assumptions: the cooperator in group *k* will give a benefit *b*_*A*_ to a player in group A and *b*_*B*_ in a player in group B, where, (i) *b*_*kk*_ = *b*_*A*_ + *b*_*B*_ or (ii) *b*_*kk*_ = *b*_*A*_ = *b*_*B*_. If the benefit is a divisible good such as a product or money, it should be divided and (i) can be applied. If the benefit is an indivisible good such as a service, (ii) can be applied. Both of the cases can be covered in this model, as we shall see in the expansion of the model that the benefit itself shall disappear from the dynamics.

This continues until the terminals unless a defector is selected. If a defector is selected in group *k*, the defector only receives the benefit, *b*_*k*_, from the cooperator in the upstream group, does not complete a task to pay the cost of cooperation and does not pass the task to his downstream. Hence, the division of labour stops there, that particular task is not completed and everyone in every group bears the loss, *g*_*k*_.

Here, we explain our assumption about the losses caused by defectors. We assume that the loss to everyone if a defector is chosen in group *k* is *g*_*k*_, which is divided into the immediate branching of group *k*; $g_{k}=\sum_{\;j}g_{\;j}$ where *j*s are the immediate branching of *k*. Each player suffers from the same losses caused by defectors in the whole system. However, from the viewpoint of group *k*, the losses a player in group *k* suffers are classified into three types: the self-inflicted loss (*γ*_*k*_), the potential loss (*g*_*ok*_) and the loss caused by *N*_*k*_ (*g*_*nk*_). The total loss to a player in group *k* is *γ*_*k*_ + *g*_*ok*_ + *g*_*nk*_.

The self-inflicted loss *γ*_*k*_ is *g*_*k*_ when a defector is chosen in group *k*, and zero when a cooperator is chosen. When defection occurs in *U*_*k*_, *γ*_*k*_ = *g*_*k*_; we assume that when the downstream player suffers from the loss caused by the upstream defector, the loss of the downstream player is the same as what he would have suffered by his defection. This means, when a player in a group in *U*_*k*_ chooses defection, the product or service which would have been produced or done by the player in group *k* was not produced or done, so the loss borne by the player in group *k* is the same as *g*_*k*_.

When the player in group *k* has already cooperated, some part of *D*_*k*_, may cooperate and some part may not. Then, the cooperator in group *k* suffers from the loss by defection in *D*_*k*_. This loss is called the potential loss to cooperators in the upstream groups, *g*_*ok*_, which is defined as the combined loss through branching in *D*_*k*_; $g_{ok}=\sum \delta_{\;j}(t)g_{\;j}$ where *j* ∈ *D*_*k*_ and *δ*_*j*_(*t*) is 1 when a chosen player in group *j*s is a defector in *D*_*k*_ at time *t*, and zero otherwise. When the cooperator is in the group *k* and all the chosen players in the groups of *k*’s immediate branching are defectors, then $g_{ok}=\sum g_{\;j}=g_{k}$. Otherwise, *g*_*ok*_ < *g*_*k*_. This condition makes the choice of branching for a player impartial, as the distributed risk of defection is the same as or lower than defection by oneself.

We also assume that any player in group *k* suffers from the loss caused by *N*_*k*_, *g*_*nk*_. The value *g*_*nk*_ includes the losses caused by defectors in *N*_*k*_. In addition, when there is a defection in a group *l* ∈ *U*_*K*_, that also has branches in *N*_*k*_, the loss *g*_*l*_ damages everyone in every group. A part of this loss comes to group *k* as the self-inflicted loss *g*_*k*_, the rest of it flows in *N*_*k*_ and gives rise to the self-inflicted losses of players in groups such as those in $D_{l}\cap N_{k}$. To calculate this loss we take the summation of the self-inflicted losses of the terminals of $D_{l}\cap N_{k}$. In other words, *g*_*nk*_ also includes the self-inflicted losses of the groups which are in the terminal of *N*_*k*_ and had a defector in their upstream that intersects with *U*_*k*_. In sum, the mathematical definition: $g_{nk}=\sum g_{\;j}+g_{m}$, where *j* ∈ *N*_*k*_ and *j* are defectors when cooperators are selected in $U_{k}\cap U_{\;j}$, and *m* ∈ *N*_*k*_ and *m* are terminals when a defector is selected in $U_{k}\cap U_{m}$.

**Figure 2.**
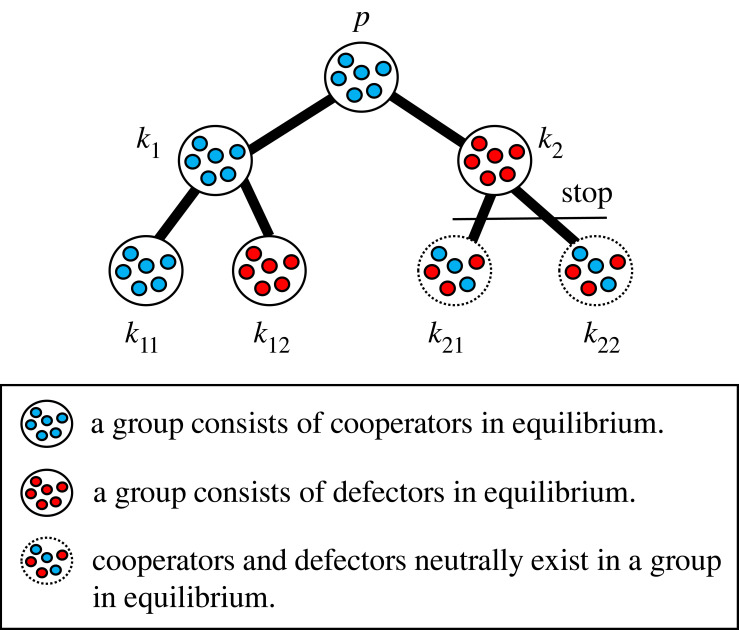
The image of a tree graph in equilibrium. The loss distribution in this system of 2-regular 2-branched directed tree graph is shown in table 2. The premier group’s name is *premier* or *p*, and the other groups' names except the premier group are *k*_*i*_s where *i*s are 1, 2, 11, 12, 21 and 22. The groups *p*, *k*_1_ and *k*_11_ are groups consisting of cooperators in equilibrium. The groups *k*_12_ and *k*_2_ are groups consisting of defectors in equilibrium. As the division of labour stops at *k*_2_, no interaction between groups occurs after *k*_2_, and the groups *k*_21_ and *k*_22_ are groups where cooperators and defectors neutrally exist. The relative equilibria in this case from the perspective of each group are also enlisted in table 2.

Figure 2 and table 2 show the example of the losses in the 2-regular 2-branched directed tree graph. Here if the group index is *k*_*j*_, the loss is shown as $g_{k_{\;j}}$. The relationship between losses is *g*_*p*_ = $g_{k_{1}}+g_{k_{2}}$, $g_{k_{1}}=g_{k_{11}}+g_{k_{12}}$, $g_{k_{2}}=g_{k_{21}}+g_{k_{22}}$. The self-inflicted loss of a cooperator in the premier group is zero. The potential loss that a cooperator in the premier group suffers from defection in the downstream groups, *D*_*p*_, is *g*_*op*_, which is the sum of the losses when defectors are selected after the premier group, $g_{op}=g_{k_{12}}+g_{k_{2}}$ in figure 2. *g*_*np*_ is zero because *N*_*p*_ is empty. In group *k*_11_, the self-inflicted loss is zero, the potential loss is zero, and $g_{nk_{11}}$ is $g_{k_{12}}+g_{k_{2}}$ because defection occurs in both groups *k*_12_ and *k*_2_ in $N_{k_{11}}$. Table 6 shows the three types of losses of other groups in figure 2.

Based on our assumption mentioned above, we can calculate the payoff of either a cooperator or a defector in group *k* (table 1); tables 1, 3 and 4 are when *k* = *p*, *k* is neither *p* nor terminal, and *k* = *n* is a terminal, respectively. The parameters are shown in table 5.

**Table 3.** The payoff matrix in the baseline system for *k* ≠ *p*.

cases	all being cooperator in **O**_ *k*_	a defector in *U*_*k*_	a defector in *D*_*k*_
*k* cooperator	*b*_*k*_ − *x*_*k*_ − *g*_*nk*_	−*g*_*k*_ − *g*_*nk*_	*b*_*k*_ − *x*_*k*_ − *g*_*ok*_ − *g*_*nk*_
*k* defector	*b*_*k*_ − *g*_*k*_ − *g*_*nk*_	−*g*_*k*_ − *g*_*nk*_	*b*_*k*_ − *g*_*k*_ − *g*_*nk*_

**Table 4.** The payoff matrix in the baseline system for *n* ≠ *p* in terminal.

cases	all being cooperator in **O**_ *n*_	a defector in *U*_*n*_
*n* cooperator	*b*_*n*_ − *x*_*n*_ − *g*_*nn*_	−*g*_*n*_ − *g*_*nn*_
*n* defector	*b*_*n*_ − *g*_*n*_ − *g*_*nn*_	−*g*_*n*_ − *g*_*nn*_

**Table 5.** Parameters.

**G**	set of nodes in the tree graph
*k*	index of nodes
*p*	index of the first or original node, called the premier
*O*_*k*_	set of nodes which create *k*’s connection with the premier and are present on the branches of *k*
*N*_*k*_	$G-(O_{k}\cup \{k\})$
*U*_*k*_	set of the upstream nodes of *O*_*k*_ with respect to *k*
*D*_*k*_	set of the downstream nodes of *O*_*k*_ with respect to *k*
*c*_*ok*_	probability of all the players in all the groups of **O**_ *k*_
*d*_*uk*_	probability of a defector in *U*_*k*_
*d*_*dk*_	probability of defector(s) in the downstream of group *k* in *D*_*k*_
*g*_*k*_	loss to everybody if a defector is chosen in group *k*
*γ*_*k*_	the self-inflicted loss in group *k*
*g*_*ok*_	the potential loss in *D*_*k*_
*g*_*nk*_	the loss caused by *N*_*k*_
*x*_*k*_	cooperation cost of a player in group *k*
*b*_*k*_	benefit of a player in group *k*
*k*_*c*_	the frequency of cooperator in group *k*
*k*_*d*_	the frequency of defector in group *k*
*f*	amount of punishment
*ρ*	probability of catching a defector, *ρ* is considerably low

### 2.2. Replicator equations for asymmetric games

If we assume that each player imitates the behaviour of others with a higher payoff in the same group, we can apply the replicator equations for asymmetric games. To calculate the expected payoff of players in the replicator equations of asymmetric games, three parameters of probability are defined; $c_{ok}=\Pi_{i\in O_{k}}i_{c}$ is the probability of all the selected players in all the groups of *O*_*k*_ being cooperators, where *i*_*c*_ is the frequency of cooperators, and *i*_*d*_ is the frequency of defectors in the group *i*. Here, *i*_*c*_ + *i*_*d*_ = 1. $d_{uk}=1-\Pi_{i\in U_{k}}i_{c}$ is the probability of a defector being selected in the groups of *U*_*k*_. $d_{dk}=(1-\Pi_{i\in D_{k}}i_{c})(\Pi_{i\in U_{k}}i_{c})$ is the probability of defector(s) being selected in the groups which are downstream of *k*, or in other words in *D*_*k*_ there is a defector or defectors. We find, *c*_*ok*_ + *d*_*uk*_ + *d*_*dk*_ = 1.

When *k* is the premier, if the player from *k* is a cooperator, and all the players who are selected from other groups are also cooperators, then the payoff of the player in *k* is *b*_*p*_ − *x*_*p*_, as they receive the benefit *b*_*p*_ and pay the cost of cooperation *x*_*p*_, to produce a product or do a task (table 1). If there are one or more defectors in *p*’s downstream then the payoff is *b*_*p*_ − *x*_*p*_ − *g*_*op*_, as the combined loss due to defection *g*_*op*_ will also be borne (table 1). Therefore, when the player from the premier group is a cooperator, then their expected payoff, $\Pi_{cp}$, is *c*_*op*_(*b*_*p*_ − *x*_*p*_) + (1 − *c*_*op*_)(*b*_*p*_ − *x*_*p*_ − *g*_*op*_) (table 1). If the player from the premier group is a defector then their expected payoff, $\Pi_{dp}$, is *b*_*p*_ − *g*_*p*_ (table 1) as he receives the benefit *b*_*p*_ but does not pay the cost of cooperation. However, due to his defection he needs to bear the loss *g*_*p*_. As he is in the premier group, his defection leads to the linear division of labour being stopped. So, the latter groups’ player’s strategy does not have any effect on his payoff, when he is a defector. Therefore, the replicator equation of a cooperator in the premier group is,

2.1 $$\frac{dp_{c}}{dt}=p_{c}(1-p_{c})(\Pi_{cp}-\Pi_{dp})=p_{c}(1-p_{c})(c_{op}g_{op}+g_{\;p}-g_{op}-x_{\;p}).$$

When *k* is neither the premier nor a terminal, if the player from *k* is a cooperator, and all the players who are selected from other groups in *O*_*k*_ are also cooperators, then the payoff of the player in *k* is *b*_*k*_ − *x*_*k*_ − *g*_*nk*_, as they receive the benefit *b*_*k*_, pay the cost of cooperation *x*_*k*_, and also bear the loss *g*_*nk*_ due to the possible defections in *N*_*k*_ (table 3). If there are one or more defectors in *k*’s downstream then the payoff is *b*_*k*_ − *x*_*k*_ − *g*_*ok*_ − *g*_*nk*_, as the combined loss due to defection *g*_*ok*_ will also be borne (table 3). If there is a defector in the *U*_*k*_, then the task does not reach *k*, so in that case he only bears the loss *g*_*k*_ and their payoff becomes −*g*_*k*_ − *g*_*nk*_. So, the expected payoff of a cooperator in group *k*, $\Pi_{ck}$, is *c*_*ok*_(*b*_*k*_ − *x*_*k*_) − *d*_*uk*_*g*_*k*_ + *d*_*dk*_(*b*_*k*_ − *x*_*k*_ − *g*_*ok*_) − *g*_*nk*_. If the player from *k* is a defector and all the chosen players in *O*_*k*_ are cooperators, or there are defectors to be chosen in *D*_*k*_, then his payoff is *b*_*k*_ − *g*_*k*_ − *g*_*nk*_ as they receive the benefit *b*_*k*_ but do not pay the cost of cooperation. However, due to their defection, they need to bear the loss *g*_*k*_. If there is a defector in the *U*_*k*_, their payoff is the same as being a cooperator, because they do not get a chance to play their strategy. So, when a player in group *k* is a defector, the expected payoff, $\Pi_{dk}$, is *c*_*ok*_(*b*_*k*_ − *g*_*k*_) − *d*_*uk*_*g*_*k*_ + *d*_*dk*_(*b*_*k*_ − *g*_*k*_) − *g*_*nk*_. Therefore, the replicator equation of a cooperator in group *k* is,

2.2 $$\frac{dk_{c}}{dt}=k_{c}(1-k_{c})(\Pi_{ck}-\Pi_{dk})=k_{c}(1-k_{c})\{c_{ok}g_{ok}+(1-d_{uk})(g_{k}-x_{k}-g_{ok})\}.$$

When *n* is a terminal group, the division of labour is effectively a linear network from the point of view of *n*. However, the aspect of possible defections in *N*_*n*_ needs to be considered. If the chosen player from *n* is a cooperator and players chosen from all the other groups in *O*_*k*_ are also cooperators, then their payoff is *b*_*n*_ − *x*_*n*_ − *g*_*nn*_. If there is a defector chosen in any of the groups of *O*_*n*_, then the task does not reach *n*, so their payoff in the terminal group becomes −*g*_*n*_ − *g*_*nn*_, regardless of them being a cooperator or a defector. When all the players chosen from all the other groups in *O*_*n*_ are cooperators, however, the player chosen in *n* is a defector, then his payoff is *b*_*n*_ − *g*_*n*_ − *g*_*nn*_, as he will have to bear the loss, *g*_*n*_ due to his own defection (table 4).

Hence, a cooperator’s expected payoff from a terminal group *n*, $\Pi_{cn}$ is *c*_*on*_(*b*_*n*_ − *x*_*n*_) − *d*_*un*_*g*_*n*_ − *g*_*nn*_. When the player from the terminal *n* is a defector, his expected payoff, $\Pi_{dn}$ is *c*_*on*_(*b*_*n*_ − *g*_*n*_) − *d*_*un*_*g*_*n*_ − *g*_*nn*_. Therefore, the replicator equation of a cooperator in the terminal group is,

2.3 $$\frac{dn_{c}}{dt}=n_{c}(1-n_{c})(\Pi_{cn}-\Pi_{dn})=n_{c}(1-n_{c})\{c_{on}(g_{n}-x_{n})\}.$$

Here, the benefit *b*_*k*_ given by a cooperator of the upstream as well as the term *g*_*nk*_ which represents the loss caused by *N*_*k*_ are both cancelled in equations (2.1)–(2.3). Therefore, they do not have any effect on the dynamics. We will show that two values, *b*_*k*_ and *g*_*nk*_, are also cancelled out when the sanction system is introduced in the equations (§3 and appendices B and C).

### 2.3. Results

**Figure 3.**
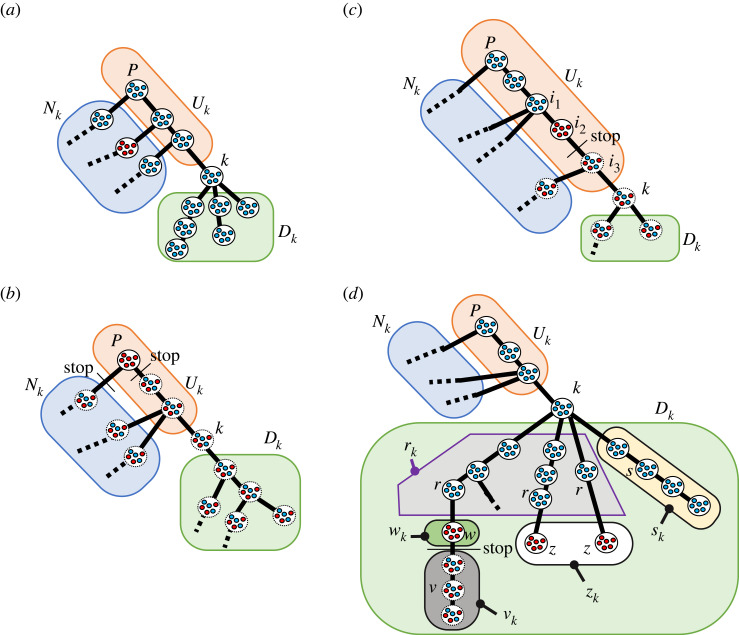
The equilibria in the system from the perspective of focal group *k*. Panel (*a*) is showing the *allc*_*k*_, (*b*) is showing the *premierD*, (*c*) is showing the ${mix}_{U_{k}\cup \{k\}}$ and (*d*) is showing the ${mix}_{D_{k}}.$

When we consider a system of 2-regular 2-branched directed tree graph such as figure 2 for example, there are 26 possible equilibrium points and we have to calculate the local stability of each of 26 equilibrium points. If we consider a larger system, the local stability of numerous possible equilibrium points should be calculated. Besides doing it, we propose a new method; the stability analysis of three sorts of equilibrium for each $O_{k}\cup \{k\}$ (figure 3).

The all-cooperation equilibrium is defined as that everyone in every group is a cooperator in $O_{k}\cup \{k\}$, which is hereafter called *allc*_*k*_ (figure 3*a*). The premier group defection equilibrium is defined as that everyone in the premier group *p* is a defector, hereafter called *premierD* (figure 3*b*). If all of the members in the premier group are defectors, then the division of labour does not start, and the latter group’s players do not have a chance to play the game, so they remain neutral (represented with *).

The equilibria are as follows (figure 3*a*,*b*):

$${allc}_{k}={\left[i_{c}=1\right]}_{i\in O_{k}\cup \{k\}},$$

and

$$premierD={\left[p_{c}=0,i_{c}=*\right]}_{i\in G-\{p\}}.$$

Finally, there is a cooperator-defector mixed equilibrium, when some groups consist of all cooperators and some are all defectors, followed by the neutral groups in $O_{k}\cup \{k\}$. There are two types of mixed equilibrium, when considering from the point of view of *k*; (i) one defector group exists in $U_{k}\cup \{k\}$ (figure 3*c*) or (ii) there are only cooperator groups in $U_{k}\cup \{k\}$, and at least one defector group exists in *D*_*k*_ (figure 3*d*). This type of mixed equilibrium is hereafter called ${mix}_{U_{k}\cup \{k\}}$. This is represented as follows (figure 3*c*):

$${mix}_{U_{k}\cup \{k\}}={\left[p_{c}=1,i_{1c}=1,{i_{2}}_{c}=0,{i_{3}}_{c}=*\right]}_{i_{2}\in U_{k}\cup \{k\}-\{p\}\;\mathrm{and}\;i_{1}\in U_{i_{2}}\;\text{and}\;i_{3}\in D_{i_{2}}}.$$

Figure 3*c* shows that $U_{k}\cup \{k\}$ is similar to the linear division of labour [56]; if someone chooses defection in a particular group, the task does not get passed to the next group, so the division of labour is stopped, and the later groups’ strategy does not matter.

The second type of mixed equilibrium, (ii), is called ${mix}_{D_{k}}$. Because of the presence of the branching in *D*_*k*_, we generalize this equilibrium as follows (figure 3*d*):

$${mix}_{D_{k}}=[{u_{1}}_{c},\ldots ,{u_{n1}}_{c},k_{c},{s_{1}}_{c},\ldots ,{s_{n2}}_{c},{r_{1}}_{c},\ldots ,{r_{n3}}_{c},{w_{1}}_{c},\ldots ,{w_{n4}}_{c},{v_{1}}_{c},\ldots ,{v_{n5}}_{c},{z_{1}}_{c},\ldots ,{z_{n6}}_{c}],$$

where, *u*_*i*__*c*_ = 1, *k*_*c*_ = 1, *s*_*i*__*c*_ = 1, *r*_*i*__*c*_ = 1, *w*_*i*__*c*_ = 0, *v*_*i*__*c*_ = *, *z*_*i*__*c*_ = 0 where *u*_*i*_ ∈ *U*_*k*_, *s*_*i*_ ∈ *s*_*k*_, *r*_*i*_ ∈ *r*_*k*_, *w*_*i*_ ∈ *w*_*k*_, *v*_*i*_ ∈ *v*_*k*_ and *z*_*i*_ ∈ *z*_*k*_. We define *u*_1_ = *p*. The mutually disjoint sets *s*_*k*_, *r*_*k*_, *w*_*k*_, *v*_*k*_ and *z*_*k*_ are defined as follows:

$$s_{k}\;:=\{s\in D_{k}|m\in O_{s}\cup \{s\}\Rightarrow m_{c}=1\},$$

$$r_{k}\;:=\{r\in D_{k}|m_{1}\in U_{r}\cup \{r\}\Rightarrow {m_{1}}_{c}=1,\;\mathrm{and}\;\exists m_{2}\in D_{r},\;\mathrm{where}\;{m_{2}}_{c}=0\},$$

$$w_{k}\;:=\{w\in D_{k}\;\mathrm{and}\;\mathrm{not}\;a\;\mathrm{terminal}|m\in U_{w}\Rightarrow m_{c}=1,\;\mathrm{and}\;w_{c}=0\},$$

$$v_{k}\;:=\{v\in D_{k}|\exists m\in U_{v}\;\mathrm{where}\;m\in w_{k}\},$$

$$z_{k}\;:=\{z\in D_{k}\;\mathrm{and}\;a\;\mathrm{terminal}|m\in U_{z}\Rightarrow m_{c}=1,\;\mathrm{and}\;z_{c}=0\},$$

where |*U*_*k*_| = *n*_1_, |*s*_*k*_| = *n*_2_, |*r*_*k*_| = *n*_3_, |*w*_*k*_| = *n*_4_, |*v*_*k*_| = *n*_5_ and |*z*_*k*_| = *n*_6_. Also,

$$s_{k}\cup r_{k}\cup w_{k}\cup v_{k}\cup z_{k}=D_{k}.$$

In simple terms, *s*_*k*_ is the set of the groups that are in *D*_*k*_, and their respective trees of the division of labour have a fully cooperative population in all the groups. *r*_*k*_ is the set of the groups in *D*_*k*_, which have fully cooperators, and the upstream groups of their respective trees of the division of labour have fully cooperators. However, at least one group has fully defectors in their downstream. *w*_*k*_ is the set of the groups that are in *D*_*k*_, not terminals, and have a full defector population. *v*_*k*_ is the set of the groups that are in *D*_*k*_ and have a fully defective group in their upstream. The strategies of the members of these groups are neutral, which is represented by * as explained before. *z*_*k*_ is the set of the terminal groups that are in *D*_*k*_, and have a full defector population.

When *k* is the premier, *p*, the ${mix}_{U_{\;p}\cup \{p\}}$ equilibrium does not exist, because there is no *U*_*p*_ and the case of *p* being the defector group is included in the equilibrium *premierD*. When *k* is a terminal, we can consider $O_{k}\cup \{k\}$ as a linear division of labour [56], where ${mix}_{D_{k}}$ does not exist.

If *k* = *p*, then $O_{\;p}\cup \{p\}=G$, in other words all the groups are in it. If we consider a certain *k*, which is not the premier, equations (2.1)–(2.3) show that *N*_*k*_ does not have any effect in the dynamics, therefore, it is enough to only consider the stability of the equilibria across $O_{k}\cup \{k\}$.

The local stability of each equilibrium is analysed (for calculations, refer to appendix A). Because the equilibria in the general tree graph are hard to write individually, we consider an arbitrary group *k* and define the classes of the equilibria while focusing on that group. The results are mentioned in table 7 in appendices A, B and C. To determine the stability of the whole system, we need to consider each of the groups individually and obtain the locally stable conditions of the respective equilibria of those individual focus groups and finally combine their conditions to obtain the locally stable state of the whole system. We shall explain this in detail in the following section using a specific example.

To study a social dilemma situation in the baseline, we consider *g*_*i*_ < *x*_*i*_ for all group *i*. Moreover, if *g*_*i*_ is high enough, it is natural that cooperation among all groups can evolve, and then we do not consider *g*_*i*_ > *x*_*i*_. With this social dilemma condition we summarize the results in table 6, which shows that *premierD* is the only stable equilibrium in the baseline, because *g*_*p*_ − (1 − *c*_*op*_)*g*_*op*_ < *x*_*p*_ is always held (table 7).

**Table 6.** Local stability conditions when *k* is the focal group in a social dilemma.

equilibrium	baseline	defector sanction	premier sanction
*premierD*	always stable	*g*_*p*_ − (1 − *c*_*op*_)*g*_*op*_ + *ρf* < *x*_*p*_	*g*_*p*_ − (1 − *c*_*op*_)*g*_*op*_ + *c*_*op*_*f* < *x*_*p*_
*allc*_*k*_	always unstable	*g*_*i*_ + *ρf* > *x*_*i*_	always unstable
		where $i\in O_{k}\cup \{k\}$.	
${mix}_{U_{k}\cup \{k\}}$	always unstable	*g*_*i*_ − *g*_*oi*_ + *ρf* > *x*_*i*_,	always unstable
		*g*_*j*_ − (1 − *c*_*oj*_)*g*_*oj*_ + *ρf* < *x*_*j*_,	
		when *k* is not a terminal.	
		*g*_*i*_ − *g*_*oi*_ + *ρf* > *x*_*i*_,	
		and *g*_*j*_ − (1 − *c*_*oj*_)*g*_*oj*_ + *ρf* < *x*_*j*_,	
		when *k* is a terminal and *j* ≠ *k*.	
		*g*_*i*_ − *g*_*oi*_ + *ρf* > *x*_*i*_,	
		and *g*_*j*_ + *ρf* < *x*_*j*_,	
		when *k* is a terminal and *j* = *k*.	
		where $j\in U_{k}\cup \{k\}$,	
		*j* is the defector group and	
		*i* ∈ *U*_*j*_.	
${mix}_{D_{k}}$	always unstable	$g_{i_{1}}-g_{oi_{1}}+\rho f>x_{i_{1}}$,	always unstable
		and $g_{i_{2}}+\rho f>x_{i_{2}}$,	
		and $g_{i_{3}}-(1-c_{oi_{3}})g_{oi_{3}}+\rho f<x_{i_{3}}$	
		and $g_{i_{4}}+\rho f<x_{i_{4}}$	
		here $i_{1}\in U_{k}\cup \{k\}\cup r_{k}$,	
		*i*_2_ ∈ *s*_*k*_, *i*_3_ ∈ *w*_*k*_ and *i*_4_ ∈ *z*_*k*_	

## 3. Two sanction systems

We introduce two types of sanction systems following Nirjhor & Nakamaru [56]. One is called the defector sanction system, where the exact defector is caught and sanctioned with the amount *f*. The finding probability of the exact defector is *ρ*. The other is called the premier sanction system, where if the defection is present, whoever defects, the player in the premier group is always sanctioned with the amount *f*. We study the evolution of cooperation in the system without punishment named the baseline system and then compare its result with the two systems with sanction. The two sanction systems are also compared with each other, to find their effectiveness.

Appendices B and C show the replicator equations for asymmetric games and the results of the local stability analysis of the defector sanction system and the premier sanction system, respectively. Tables 5 and 7 summarize the local stability condition for each of the equilibrium points.

We find that *premierD* is the only stable equilibrium in the premier sanction system as well as in the baseline (tables 6 and 7), although the condition is somehow less strict for *allc* to be stable because of the punishment, the same conclusion can be drawn here as well as the baseline. In the defector sanction system, however, all the equilibria are conditionally stable.

**Figure 4.**
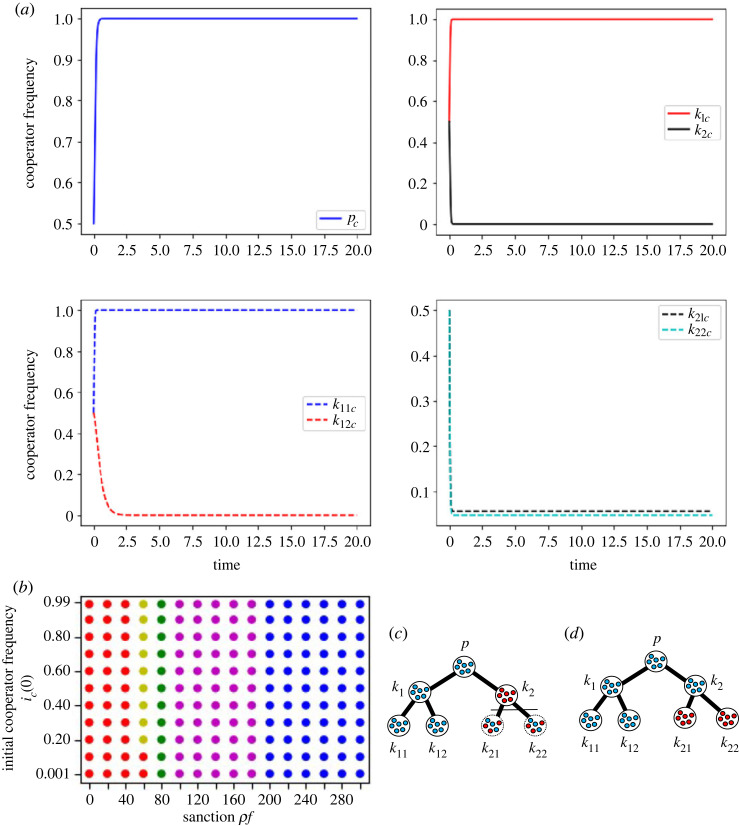
(*a*) The numerical simulation outcomes in a 2-regular 2-branched network as shown in figure 2 when the *i*_*c*_(0) = 0.5 and *ρf* = 58, and the evolutionary dynamics converges to one of states such as figure 2. In each graph of (*a*), the horizontal axis is for time and the vertical axis is for the frequency of cooperators in each group. The left-upper graph shows the dynamics in the premier group; the right-upper graph, group *k*_1_ (red) and group *k*_2_ (black); the left-lower, the group *k*_11_ (blue dashes) and group *k*_12_ (red dashes); the right-lower, the group *k*_21_ (black dashes) and group *k*_22_ (light blue dashes). (*b*) Presents the effect of sanction, *ρf* in the defector sanction system, and the initial frequency of cooperators, *i*_*c*_(0), on the simulation outcomes. In (*b*), yellow, green and magenta dots present three types of mixed equilibria, presented by figure 2, figure 4*c*,*d*, respectively. The red and blue dots represent the *premierD* and *allc* equilibria, respectively. The parameters are: *g*_*p*_ = 64, $g_{k_{1}}=g_{k_{2}}=32$, $g_{k_{11}}=g_{k_{12}}=g_{k_{21}}=g_{k_{22}}=16$, *x*_*p*_ = 65, $x_{k_{1}}=35$, $x_{k_{2}}=99$, $x_{k_{11}}=20$, $x_{k_{12}}=77$, $x_{k_{21}}=207$, $x_{k_{22}}=215$.

We would like to explain the equilibrium using figures 2 and 4*a*. When a defector-sanction system is applied, figure 2 is the image of the equilibrium and figure 4*a* is the time-change of the frequencies of cooperators in each group. To obtain the local stability condition for this whole system, **G**, we do the local stability analysis for the equilibrium point with respect to *k* without considering the effect of *N*_*k*_, using equations (1)–(3). In the case of figures 2 and 4*a*, where **G** converges to the ${mix}_{D_{\;p}}$ equilibrium, we can obtain the local stability condition of the ${mix}_{D_{\;p}}$ equilibrium in the whole system **G**. Additionally, the $allc_{k_{11}}$ equilibrium with respect to *k*_11_ should be locally stable, the ${mix}_{U_{k_{i}}\cup \{k_{i}\}}$ equilibrium with respect to *k*_*i*_ where *i* is 2, 12, 21, 22 and the ${mix}_{D_{k_{1}}}$ equilibrium with respect to *k*_1_ should be locally stable (figures 2 and 4*a*). The stable equilibria from the perspective of different groups are included in table 2.

In figure 4*a*, as *x*_*p*_ − *g*_*p*_ + (1 − *c*_*op*_)*g*_*op*_ = 49 < 58 = *ρf*, the premier group becomes full cooperator. As $x_{k_{1}}-g_{k_{1}}+(1-c_{ok_{1}})g_{ok_{1}}=19<58=\rho f$ and $x_{k_{2}}-g_{k_{2}}+(1-c_{ok_{2}})g_{ok_{2}}=97.27>58=\rho f$, *k*_1_ becomes full cooperator, and *k*_2_ becomes full defector, which in return makes *k*_21_ and *k*_22_ neutral. $x_{k_{11}}-g_{k_{11}}=4<58=\rho f$ and $x_{k_{12}}-g_{k_{12}}=61>58=\rho f$, which means *k*_11_ becomes full cooperator and *k*_12_ becomes full defector (table 5). For this reason, with respect to group *k*_11_, the groups in $O_{k_{11}}\cup \{k_{11}\}$ converges to $allc_{k_{11}}$. With respect to *k*_12_, the groups in $O_{k_{12}}\cup \{k_{12}\}$ converge to the ${mix}_{U_{k_{12}}\cup \{k_{12}\}}$ equilibrium and are not influenced by the groups in $N_{k_{12}}$.

Figure 4*b* shows the outcomes of the numerical simulations when costs are determined randomly. When sanction is small, the simulations converge to the *premierD* equilibrium (red); when sanction is large enough, the simulations converge to the *allc* equilibrium (blue). Between *premierD* and *allc* equilibria, the dynamics converges to the mixed equilibrium point shown in figure 2 (yellow). When we change the value of sanction, we obtain other mixed equilibria presenting figure 4*c*,*d* shown with green and magenta dots, respectively.

To understand the dynamics more concretely, we do some simplification and numerical analysis in the following section.

## 4. Numerical analysis

Firstly, we consider a special case; a *κ*-regular, *μ*-branched directed finite graph is assumed, for making numerical analysis. When *j*s are the immediate branching of *k*, $g_{k}=\sum_{\;j}g_{\;j}$. In this case, we also consider the distribution of the loss is uniform in each branching. If *k* is at the *μ*_*k*_th branch (where 0 ≤ *μ*_*k*_ ≤ *μ*), we assume, $g_{k}={\left(1/\kappa \right)}^{\mu_{k}}g.$

For the 2-regular, 2-branched graph, the expected values of *g*_*op*_, $g_{ok_{1}}$, and $g_{ok_{2}}$, *E*[*g*_*op*_], $E[g_{ok_{1}}]$ and $E[g_{ok_{2}}]$, are as follows (figure 2): $E[g_{op}]=g_{k_{1}}(1-k_{1c})+k_{1c}E[g_{ok_{1}}]+g_{k_{2}}(1-k_{2c})+k_{2c}E[g_{ok_{2}}]$, where $E[g_{ok_{1}}]=$ $g_{k_{11}}(1-k_{11c})+g_{k_{12}}(1-k_{12c})$, $E[g_{ok_{2}}]=g_{k_{21}}(1-k_{21c})+g_{k_{22}}(1-k_{22c})$, and *k*_22*c*_, for example, is the frequency of cooperators in group *k*_22_ (table 4).

**Figure 5.**
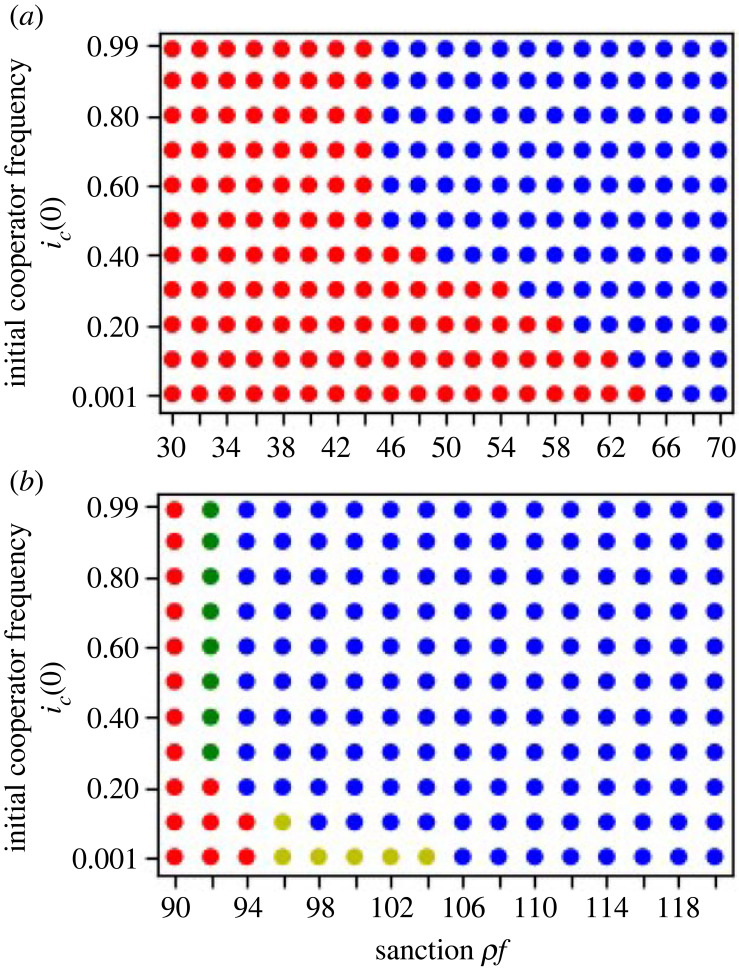
Evolutionary dynamics in the 2-regular 2-branched directed finite graph when (*a*) the cost of the cooperation decreases downstream, and (*b*) when the cost of the cooperation increases downstream. The blue dot represents when the system converges to *allc* equilibrium, or everyone in every group is a cooperator. The red dots represent the simulation dynamics converged to the *premierD* equilibrium, the yellow dot represents the mixed equilibrium when the premier group is full cooperator and the groups in the first branching or *k*_1_ and *k*_2_ are full defectors, the green dot represents the mixed equilibrium when the groups premier, *k*_1_ and *k*_2_ are full cooperators and the groups in the second branching *k*_11_, *k*_12_, *k*_21_ and *k*_22_ are full defectors. (*a*) The bistability between the red-blue bistability, and (*b*) shows the red-blue bistability, red-green and yellow-blue co-stability under the same sanction. Both of the figures show that sanction promotes the evolution of cooperation. The stable existence of mixed equilibrium in (*b*) represents the stable co-existence of fully cooperator and fully defector groups in the same network when sanction is applied. The parameters in (*a*) are: *g*_*p*_ = 64, $g_{k_{1}}=g_{k_{2}}=32$, $g_{k_{11}}=g_{k_{12}}=g_{k_{21}}=g_{k_{22}}=16$, *x*_*p*_ = 65, $x_{k_{1}}=x_{k_{2}}=63$, $x_{k_{11}}=x_{k_{12}}=x_{k_{21}}=x_{k_{22}}=61$. The parameters in (*b*) are: *g*_*p*_ = 92, $g_{k_{1}}=g_{k_{2}}=46$, $g_{k_{11}}=g_{k_{12}}=g_{k_{21}}=g_{k_{22}}=23$, *x*_*p*_ = 95, $x_{k_{1}}=x_{k_{2}}=105$, $x_{k_{11}}=x_{k_{12}}=x_{k_{21}}=x_{k_{22}}=115$.

Figure 5 shows the effect of *ρf* on the dynamics in the 2-regular 2-branched directed finite graph. For simplicity, we consider the cost of the cooperation for the groups which are present in the same level branching to be the same.

Figure 5*a* is the results of the numerical analysis of equations (B 1)–(B 3) in appendix B when the cost of cooperation decreases downstream under the defector sanction system. Figure 5*a* shows that our numerical simulation results match our theoretical prediction (table 6). In figure 5*a*, the simulation dynamics converges to the *premierD* equilibrium in *ρf* < 66, which matches *ρf* < *x*_*p*_ where all groups are almost full of defectors, *c*_*op*_ ≈ 0, and *g*_*op*_ = *g*_*p*_, and the dynamics also converges to the *allc* equilibrium in *ρf* ≥ 46 starting from a very high initial value of *i*_*c*_(0), which matches *ρf* + *g*_*i*_ > *x*_*i*_ for all the group *i*s. The initial frequencies determine which dynamics converge to the *premierD* or *allc* equilibria in *ρf* between 46 and 64.

Figure 5*b* shows the numerical simulation outcomes when the cost of the cooperation increases downstream. The simulation dynamics converges to the *premierD* in *ρf* ≤ 94 which matches *ρf* + *g*_*p*_ − (1 − *c*_*op*_)*g*_*op*_ < *x*_*p*_ where all groups are almost full of defectors, *c*_*op*_ ≈ 0, and *g*_*op*_ = *g*_*p*_ (table 6). When *ρf* is 92 in figure 5*b*, there is a co-presence of green dots and red dots, which indicates the stability predicted by table 6; the initial frequency of cooperators in groups determines if the dynamics converge to either the mixed equilibrium ${mix}_{D_{\;p}}$ or the *premierD*. When *ρf* ≥ 94, the simulation converges to the *allc* equilibrium starting from the almost full cooperator groups, which can be predicted by *g*_*i*_ + *ρf* > *x*_*i*_ for all group *i*s (table 6). The red dots and the blue dots are co-present when *ρf* is 94 in figure 5*b*, which matches the theoretical prediction; the bistability between the equilibria *premierD* and *allc*. The numerical simulations can show the co-presence of blue and yellow dots when *ρf* is from 96 to 104 in figure 5*b*.

**Figure 6.**
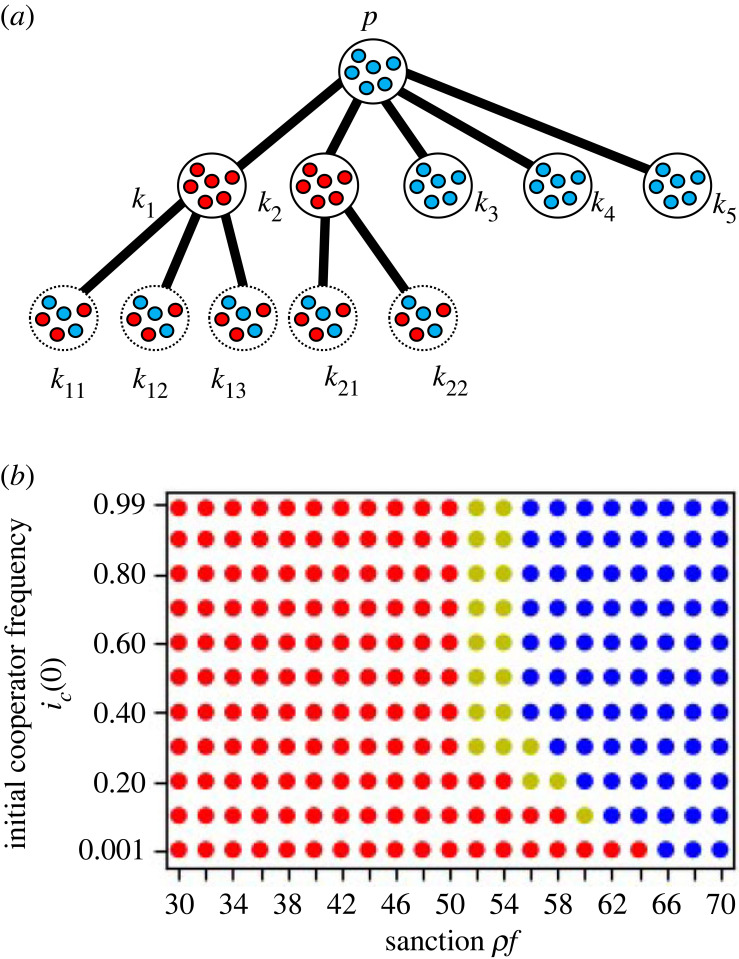
(*a*) The network structure which imitates the bureaucratic structure of the US Department of State, and one of equilibrium states. (*b*) The effect of sanction, *ρf*, and the initial frequency of cooperators on the numerical simulation outcomes. The red and blue dots represent the *premierD* and *allc* equilibria, respectively. The yellow dot presents the dynamics converging to the equilibrium shown in (*a*). Even though the cost is decreasing downstream, the simulation converges to the mixed equilibrium presented by (*a*) in (*b*). The parameters, which are given irrelevant to the bureaucratic structure, are: *x*_*p*_ = 65, $x_{k_{1}}=x_{k_{2}}=x_{k_{3}}=x_{k_{4}}=x_{k_{5}}=63$, $x_{k_{11}}=x_{k_{12}}=x_{k_{13}}=59$, $x_{k_{21}}=x_{k_{22}}=61$, *g*_*p*_ = 60, $g_{k_{1}}=g_{k_{2}}=g_{k_{3}}=g_{k_{4}}=g_{k_{5}}=12$, $g_{k_{11}}=g_{k_{12}}=g_{k_{13}}=4$, $g_{k_{21}}=g_{k_{22}}=6$.

We have only considered the evolutionary dynamics in the symmetric tree network. In reality, there are division of labour in asymmetric tree networks. The network in figure 6*a* is inspired by the simplest tree-like bureaucratic structure of the US Department of State shown in the 15th chapter of American Government (2e Second Edition) (2019) by Openstax and Lumen Learning [57]. The network has one premier node as the secretary of state, then 23 nodes branched in the first level of branching, and 7 of them have branching of themselves in another level of branching. We consider the ratio of it and take a simpler network with a similar ratio for our numerical analysis. We take a network in which in the first level of branching, 5 branches come out of the premier node, then two of them have further branching, one has 2 branches and one has 3 branches in figure 6*a*.

The numerical simulation outcomes are shown in figure 6*b* where costs decrease downstream. Table 5 indicates that the mixed equilibria can also be locally stable when the cost of the cooperation decreases downstream. This is because *g*_*i*_ − *g*_*oi*_ + *ρf* > *x*_*i*_ > *x*_*j*_ > *g*_*j*_ − (1 − *c*_*oj*_)*g*_*oj*_ + *ρf* as well as *g*_*i*_ − *g*_*oi*_ + *ρf* > *x*_*i*_ > *x*_*j*_ > *g*_*j*_ + *ρf* have no contradiction when *j* ∈ *D*_*i*_ (table 6). Our numerical simulations can also show that the dynamics converge to the mixed equilibrium point (yellow dots in figure 6*b*). While the mixed equilibrium point cannot be locally stable and no numerical simulations converged to the mixed equilibrium point in the linear division of labour when the cost decreases downstream [56], and therefore the existence of mixed equilibrium points when the cost decreases downstream is unique from this study.

## 5. The effect of the network size

**Figure 7.**
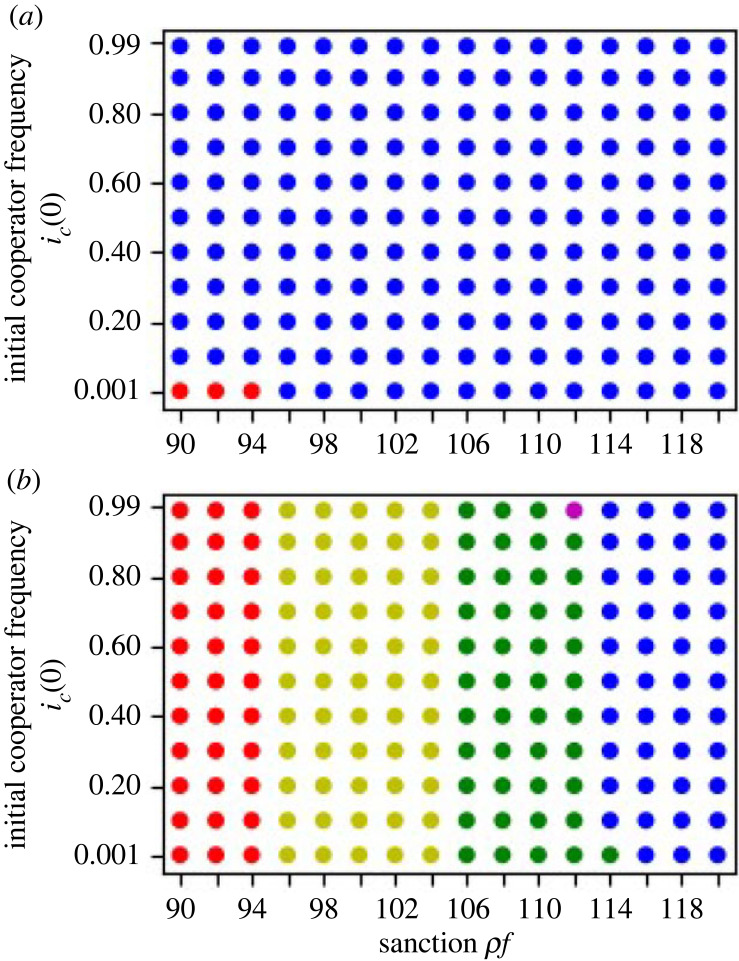
Showing the effect of adding or removing a level of branching in downstream of a network on the evolution of cooperation compared with figure 5*b*. (*a*) The numerical analysis results in a 2-regular once-branched network (figure 8*a*). (*b*) The numerical analysis results in a 2-regular thrice-branched network (figure 8*b*). The red and blue dots represent the *premierD* and *allc* equilibria, respectively. The yellow, green and magenta dots present the mixed equilibria when the first level of branching, second level of branching, and the third level of branching become the full defector groups, respectively. The parameters are in (*a*): *x*_*p*_ = 95, $x_{k_{1}}=x_{k_{2}}=105$, *g*_*p*_ = 92, $g_{k_{1}}=g_{k_{2}}=46$. The parameters in (*b*) are: *x*_*p*_ = 95, $x_{k_{1}}=x_{k_{2}}=105$, $x_{k_{11}}=x_{k_{12}}=x_{k_{21}}=x_{k_{22}}=115$, $x_{k_{111}}=x_{k_{112}}=x_{k_{121}}=x_{k_{122}}=x_{k_{211}}=x_{k_{212}}=x_{k_{221}}=x_{k_{222}}=125$, *g*_*p*_ = 92, $g_{k_{1}}=g_{k_{2}}=46$, $g_{k_{11}}=g_{k_{12}}=g_{k_{21}}=g_{k_{22}}=23$, $g_{k_{111}}=g_{k_{112}}=g_{k_{121}}=g_{k_{122}}=g_{k_{211}}=g_{k_{212}}=g_{k_{221}}=g_{k_{222}}=11.5$. See figure 8*b* for the notations.

**Figure 8.**
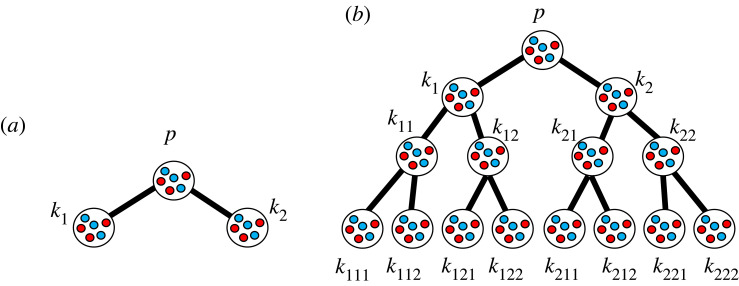
(*a*) The 2-regular once-branched tree graph and (*b*) 2-regular thrice-branched tree graph network. By adding one more level downstream, (*a*) is transformed into the 2-regular twice-branched tree graph network (figure 2), which is transformed into (*b*).

There are two ways in which a given network can increase in size. One is when more levels are added downstream of it and the other is when more branches are added to a network in parallel to other nodes. We analyse the effects of both with mathematical analysis and then evaluate the results through numerical analysis. Appendix D shows mathematically that adding more levels downstream of the same network hinders the evolution of cooperation. This is evaluated through the comparison of numerical analysis in figures 5*b* and 7*a*,*b*. Figure 7*a* shows the evolutionary dynamics converges to equilibria of the network which has one less level downstream than the network of figure 5*b*, in other words, 2-regular once-branched network (figure 8*a*), with the same cost of cooperation, and losses to the groups in each node as figure 5*b*. Figure 7*b* shows the evolutionary dynamics converges to equilibria of the network which has one more level downstream than the network of figure 5*b*; in other words, 2-regular thrice branched network (figure 8*b*), with the same cost of cooperation and losses to the groups in each node as the figure 5*b*. For simplicity and consistency with the settings in figure 5*b*, we considered that, as there is one more level of branching, the loss is uniformly divided once more and the cost is increased with the same increment as the previous levels in the network for which figure 7*b* shows the convergence of the evolutionary dynamics.

When the initial frequency of the cooperators is very low such as 0.001, as 1 − *c*_*op*_ ≃ 1 and *g*_*op*_ = *g*_*p*_, the local stability condition for *premierD* in group *p* is that *ρf* < *x*_*p*_ regardless of figures 5*b* and 7*a*,*b* (table 6). The local stability condition for *allc*_*k*_ indicates that with adding more levels downstream cooperation is less evolved (table 6). As a result, the bistable region or the region with mixed equilibria is wider in the network with more levels (figures 5*b* and 7*a*,*b*).

Appendix D also shows that the effect of adding a branch in parallel to the same network is uncertain.

## 6. Discussion and conclusion

We took a model of the division of labour in a finite tree graph and studied the effect of sanctions on it. There is a premier group and then the division of labour is branched from it. Each node of the tree graph has a group which has a role in the division of labour. The task flows from the upstream to the downstream and gets divided through the branching. If a player who is randomly selected from a group chooses defection, the division of labour stops there and everyone in every group needs to bear a loss according to their position. We compare the evolution of cooperation in the baseline system (which has no sanction) with the two sanction systems named the defector sanction system and the premier sanction system. We studied the general model and found three equilibria when the defector sanction system is applied in the social dilemma situation: (i) *premierD*, where all players in the whole premier group choose defection, (ii) *allc* where everyone in every group is a cooperator and (iii) mixed equilibrium, where the premier group consists of only cooperators, some other groups also are full of cooperators, and somewhere in the network there are one or more group/groups who have a whole population of defectors. We did the local stability analysis of these equilibria. Then, for doing numerical analysis, we considered a special case and verified the results of the general case.

The previous theoretical studies show that the cooperation evolves in a network structured population when *b*/*c* > *k*, where each node representing a single player has *k* regular links and *b* is the benefit from a cooperator and *c* is a cost of cooperation (e.g. [12]). However, in our study, the benefit to a player given by a cooperator from the upstream group is cancelled out in equations and does not have any effect on the evolution of cooperation. This means that our results cannot be summarized by the ratio of *b* and *c*. This result is the same as the linear division of labour [56]. This is because both the cooperators and defectors in a group receive the benefit from the cooperation in the upstream groups regardless of their own strategy. This can be interpreted as the salary given to an employee in this sort of division of labour has no effect on the evolution of cooperation.

The loss via defection becomes distributed in the branches as $g_{k}=\sum_{\;j\;\mathrm{are}\;\mathrm{immediate}\;\mathrm{branching}\;\mathrm{of}\;k}g_{\;j}$. The loss via defection *g*_*k*_ is subjective to the task assigned to the group *k*. Because of this setting a group *k*’s evolutionary dynamics are only affected by the action of groups in $O_{k}\cup \{k\}$, as those are the groups directly associated with the action that is assigned to *k*. If there is a defection in the upstream of *k*, the loss to *k* is the same as if the task is not being completed by a player in group *k*. A defection downstream of *k* means a part of the task assigned to group *k* is not eventually fully completed, which in turn affects the payoff of *k* as *g*_*ok*_. However, because of this setting the tasks assigned to groups present in *N*_*k*_ have no relation with the task of group *k*, and because of that, their defection does not affect the evolutionary dynamics of *k*. That is why *g*_*nk*_ is canceled out from the replicator equation for the dynamics of group *k*. This means that a group’s decisions are influenced only by that part of the network with which the nodes have a direct hierarchical connection with that particular group. In simpler terms, a group is influenced by other groups which are either in its hierarchical upstream or downstream, not the groups which are branched separately from its upstream but belong to the same network. This result can be applied to the division of labour of the government, as it shows that a corrupt/honest sector can exist independently and in a government, even when other sectors of the government are honest/corrupt.

Another main point of this study is to show how to calculate the local stability of equilibrium point in a general tree network; there are numerous possible equilibrium points in a tree network. If we calculate the local stability condition of each of all possible equilibrium points, it is tough work. In our work, (i) we categorize various equilibrium points into four types in terms of a specific node *k*, (ii) obtain the local stability of each of four types, (iii) the combination of these four types presents a specific equilibrium point and so (iv) we can obtain the local stability of the specific equilibrium point by the combination of four types. This is the contribution of our work from the viewpoint of mathematical modelling.

In our study, the defector sanction system prevails in both the evolution of cooperation and sustaining the co-existence of the fully cooperator and defector groups than the premier sanction system. However, this sanction system depends on the finding probability of the exact defector *ρ*. We do not assume that players have perception bias. However, in reality, there is perception bias. In Jiang *et al.* [36], it is stated through a human experiment that even though the subjective perception of being sanctioned is often less than the actual threat of being sanctioned, the higher threat regardless makes the population choose cooperation more. In our future studies, we will introduce a new assumption about risk perception and investigate the effects of subjective risk perception of sanctions in the evolution of cooperation in the division of labour.

Through comparison with the linear division of labour [56], we find that the mixed equilibria can be stable in the baseline as well as the premier sanction system, when we do not consider the social dilemma situation, in other words, *g*_*i*_ < *x*_*i*_ for all the group *i*s does not necessarily hold. In Nirjhor & Nakamaru [56], the mixed equilibria were unstable in the baseline and the first role sanction system regardless of social dilemma or not. We theoretically find that the mixed equilibrium can be stable even when the cost is decreasing downstream in the defector sanction system and show it with figure 6. In the linear division of labour of Nirjhor & Nakamaru [56], the mixed equilibrium is never stable when the cost is decreasing downstream in the defector sanction system. In other words, the coexistence of the full cooperator and full defector groups have more scope to the stable in the tree graph network than the linear network.

We should mention the applicability of this study to the supply chain. Our study can be applied to the multilayered subcontract (e.g. [6]), which has the tree structure assumed in our study. There are various networks among roles and stakeholders in the supply chain. In Lambert & Cooper [61], the generalized supply chain network was shown to be an uprooted tree-like one, where there is a central body that can be considered as the stem of the tree from which branches spread in both directions of the root and the shoot. From one direction, the branches merge upstream towards the central body showing many divisions of the labour merging into the completion of a single labour, and from there the branches split downstream showing the labour is being divided. Our study addresses the evolution of cooperation in the later part of the supply chain where the labour is being divided downstream. When each player is assumed to be located at each node of trees and to interact with the neighbours, the effect of the merging of networks or directed cycles on the evolution of cooperation has been investigated (e.g. [29]). As we assume that a group is located at each node of trees, our model and results would be different from the previous studies. In our future research, we wish to address the problem of the evolution of cooperation in the former part of the supply chain as well, where the division of labour merges together upstream to complete a single labour, and then extend our study to the uprooted tree-like networks.

## Figures and Tables

**Figure 9.**
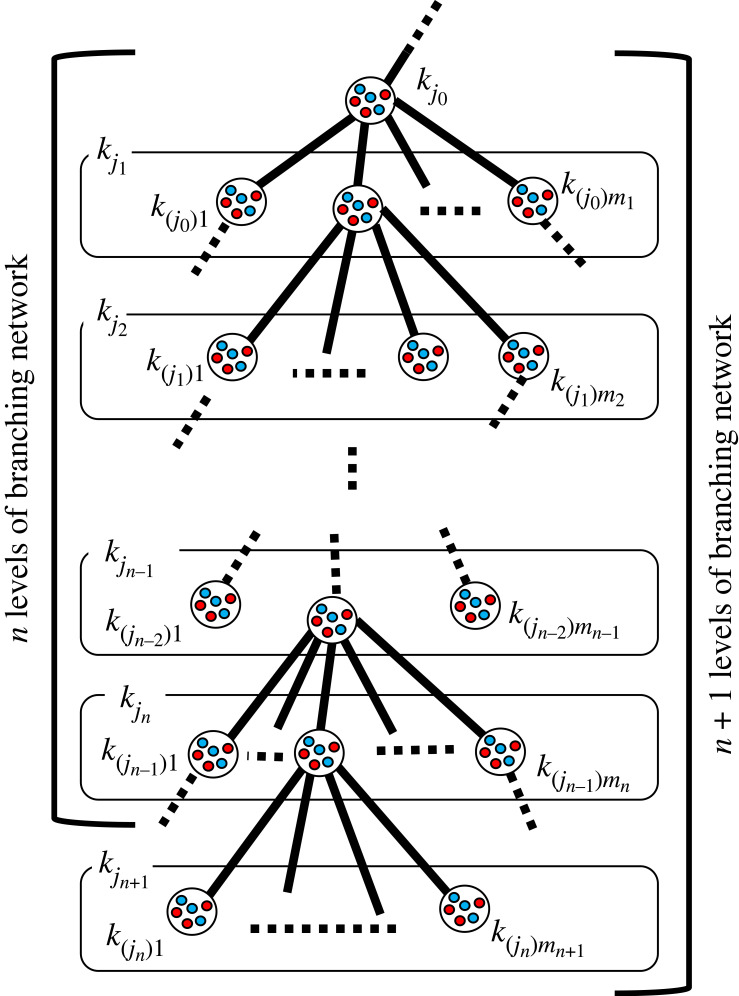
The image of the network which is used in appendix D to prove that adding levels of branching downstream of the same network hinders the evolution of cooperation. It is a tree graph network of *n* + 1 level starting from node $k_{\;j_{0}}$, which then branches into *m*_1_ branches indexed as $k_{(j_{0})1},\ldots ,\;k_{(j_{0})m_{1}}$, and these branches are summarized in the summation with index $k_{\;j_{1}}$. This indexing continues until the *n* + 1th level of branching, in other words the terminals, and the terminals are indexed as $k_{(j_{n})1},\ldots ,\;k_{(j_{n})m_{n+1}}$.

**Figure 10.**
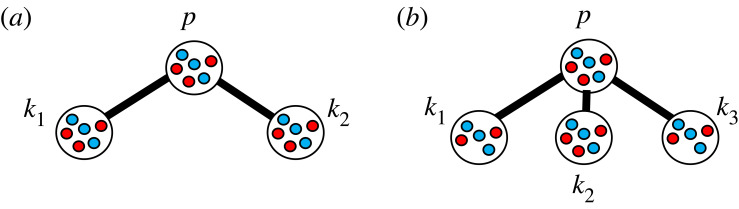
(*a*) The 2-regular once-branched tree graph and (*b*) the three regular once-branched tree graph network. (*a*) Transformed into (*b*) by adding one more branch parallel to the first level of branching.

## Data Availability

The basic MATLAB code for generating the results of this manuscript are given in the ESM file titled ‘Numerical_analysis_code_Nirjhor_and_Nakamaru_tree_graph_2023’ [62].

## Appendix A. The local stability analysis of the baseline model

On the basis of equations (2.1)–(2.3) in the main text, we make the time differential equations for $O_{k}\cup \{k\}$. Then, we calculate Jacobian matrix from the differential equations for analysing the local stability of the equilibria based on $O_{k}\cup \{k\}$. All Jacobian matrices are lower triangular matrices here, so the eigenvalues are just the main diagonal entries.

The Jacobian matrix for *premierD* is *J*(*premierD*). If *ξ* is a main diagonal component of *J*(*premierD*) then,

$$\xi =\left\{ \begin{array}{cc} g_{\;p}-x_{\;p}-(1-c_{op})g_{op},\; & \mathrm{if}\;p\;\mathrm{is}\;\mathrm{the}\;\mathrm{premier}\;\mathrm{group}\;\\ 0,\; & \mathrm{otherwise}.\; \end{array}\right.$$

When *g*_*p*_ − (1 − *c*_*op*_)*g*_*op*_ < *x*_*p*_, *PremierD* is locally stable. As *g*_*p*_, *x*_*p*_, *g*_*op*_ > 0, 0 ≤ *c*_*op*_ ≤ 1, *g*_*op*_ ≤ *g*_*p*_, and *g*_*p*_ is always lower than *x*_*p*_ in the social dilemma. Therefore, *PremierD* is always locally stable.

The Jacobian matrix for *allc*_*k*_ is *J*(*allc*_*k*_). If *ξ* is a main diagonal component of *J*(*allc*_*k*_) then

$$\xi =\left\{ \begin{array}{cc} x_{i}-g_{i},\; & \mathrm{if}\;is\;\mathrm{are}\;\mathrm{the}\;\mathrm{groups}\;\mathrm{in}\;O_{k}\;\\ 0,\; & \mathrm{otherwise}.\; \end{array}\right.$$

As the baseline model has the social dilemma situation, *x*_*i*_ > *g*_*i*_. Then, *allc*_*k*_ is not locally stable.

The Jacobian matrix for ${mix}_{U_{k}\cup \{k\}}$ is $J({mix}_{U_{k}\cup \{k\}})$. If *ξ* is a main diagonal component of $J({mix}_{U_{k}\cup \{k\}})$ and *k* is not a terminal, or *k* is a terminal however, *k* is not the defector group, then

$$\xi =\left\{ \begin{array}{cc} x_{i}-g_{i}+g_{oi},\; & \mathrm{if}\;is\;\mathrm{are}\;\mathrm{the}\;\mathrm{groups}\;\mathrm{in}\;O_{k}\;\mathrm{that}\;\mathrm{are}\;\mathrm{full}\;\mathrm{cooperators}\;\\ g_{\;j}-x_{\;j}-(1-c_{oj})g_{oj},\; & \mathrm{if}\;j\;\mathrm{is}\;\mathrm{the}\;\mathrm{group}\;\mathrm{in}\;O_{k}\;\mathrm{that}\;\mathrm{is}\;\mathrm{full}\;\mathrm{defector}\;\\ 0,\; & \mathrm{otherwise}.\; \end{array}\right.$$

If *ξ* is a main diagonal component of $J({mix}_{U_{k}\cup \{k\}})$ and *k* is a terminal and *k* is the defector group, then

$$\xi =\left\{ \begin{array}{cc} x_{i}-g_{i}+g_{oi},\; & \mathrm{if}\;is\;\mathrm{are}\;\mathrm{the}\;\mathrm{groups}\;\mathrm{in}\;O_{k}\;\mathrm{that}\;\mathrm{are}\;\mathrm{full}\;\mathrm{cooperators}\;\\ g_{\;j}-x_{\;j},\; & \mathrm{if}\;j\;\mathrm{is}\;\mathrm{the}\;\mathrm{group}\;k\;\mathrm{that}\;\mathrm{is}\;\mathrm{full}\;\mathrm{defector}\; \end{array}\right.$$

As the baseline model has the social dilemma situation, *x*_*i*_ > *g*_*i*_ − *g*_*oi*_. Therefore, the ${mix}_{U_{k}\cup \{k\}}$ is not stable.

The Jacobian matrix for ${mix}_{D_{k}}$ is $J({mix}_{D_{k}})$. If *ξ* is a main diagonal component of $J({mix}_{D_{k}})$ then

$$\xi =\left\{ \begin{array}{cc} x_{i_{1}}-g_{i_{1}}+g_{oi_{1}},\; & \mathrm{if}\;i_{1}s\;\mathrm{are}\;\mathrm{the}\;\mathrm{groups}\;\mathrm{in}\;U_{k}\cup \{k\}\cup r_{k}\;\\ x_{i_{2}}-g_{i_{2}},\; & \mathrm{if}\;i_{2}s\;\mathrm{are}\;\mathrm{the}\;\mathrm{groups}\;\mathrm{in}\;s_{k}\;\\ g_{i_{3}}-x_{i_{3}}-(1-c_{oi_{3}})g_{oi_{3}},\; & \mathrm{if}\;i_{3}s\;\mathrm{are}\;\mathrm{the}\;\mathrm{groups}\;\mathrm{in}\;w_{k}\;\\ g_{i_{4}}-x_{i_{4}},\; & \mathrm{if}\;i_{4}s\;\mathrm{are}\;\mathrm{the}\;\mathrm{groups}\;\mathrm{in}\;z_{k}\;\\ 0,\; & \mathrm{otherwise}.\; \end{array}\right.$$

This equilibrium is always unstable as the baseline model has the social dilemma situation, $x_{i_{1}}>g_{i_{1}}-g_{oi_{1}}$, because for $J({mix}_{D_{k}})$, $U_{k}\cup \{k\}$ is never an empty set. Table 7 summarizes the local stability condition of four equilibrium points in the baseline model.

## Appendix B. The defector sanction system

The payoff matrices of the defector sanction system are shown in tables 8–10. The payoffs are the same as the baseline except while choosing defection, the players are subjected to the sanction *ρf*. Premier’s payoff in the defector sanction system when he is a cooperator, $\Pi_{cp}$ is *b*_*p*_ − *x*_*p*_ − *g*_*op*_ + *c*_*op*_*g*_*op*_. Premier’s payoff in the defector sanction system when he is a defector, $\Pi_{dp}$, is *b*_*p*_ − *g*_*p*_ − *ρf*. Therefore, the replicator equation is,

$$\frac{dp_{c}}{dt}=p_{c}(1-p_{c})(\Pi_{cp}-\Pi_{dp})=p_{c}(1-p_{c})(c_{op}g_{op}+g_{\;p}+\rho f-g_{op}-x_{\;p}).(B\;1)$$

**Table 7.** Local stability conditions when *k* is the focal group.

equilibrium	baseline	defector sanction	premier sanction
*premierD*	*g*_*p*_ − (1 − *c*_*op*_)*g*_*op*_ < *x*_*p*_	*g*_*p*_ − (1 − *c*_*op*_)*g*_*op*_ + *ρf* < *x*_*p*_	*g*_*p*_ − (1 − *c*_*op*_)*g*_*op*_ + *c*_*op*_*f* < *x*_*p*_
*allc*_*k*_	*g*_*i*_ > *x*_*i*_	*g*_*i*_ + *ρf* > *x*_*i*_	*g*_*p*_ + *f* > *x*_*p*_ and *g*_*i*_ > *x*_*i*_
	where $i\in O_{k}\cup \{k\}$	where $i\in O_{k}\cup \{k\}$	where $i\in O_{k}\cup \{k\}-\{p\}$
${mix}_{U_{k}\cup \{k\}}$	*g*_*i*_ − *g*_*oi*_ > *x*_*i*_,	*g*_*i*_ − *g*_*oi*_ + *ρf* > *x*_*i*_,	*g*_*i*_ − *g*_*oi*_ > *x*_*i*_,
	and *g*_*j*_ − (1 − *c*_*oj*_)*g*_*oj*_ < *x*_*j*_,	and *g*_*j*_ − (1 − *c*_*oj*_)*g*_*oj*_ + *ρf* < *x*_*j*_,	and *g*_*j*_ − (1 − *c*_*oj*_)*g*_*oj*_ < *x*_*j*_,
	when *k* is not a terminal.	when *k* is not a terminal.	when *k* is not a terminal.
	*g*_*i*_ − *g*_*oi*_ > *x*_*i*_,	*g*_*i*_ − *g*_*oi*_ + *ρf* > *x*_*i*_,	*g*_*i*_ − *g*_*oi*_ > *x*_*i*_,
	and *g*_*j*_ − (1 − *c*_*oj*_)*g*_*oj*_ < *x*_*j*_,	and *g*_*j*_ − (1 − *c*_*oj*_)*g*_*oj*_ + *ρf* < *x*_*j*_,	and *g*_*j*_ − (1 − *c*_*oj*_)*g*_*oj*_ < *x*_*j*_,
	when *k* is a terminal	when *k* is a terminal	when *k* is a terminal
	and *j* ≠ *k*.	and *j* ≠ *k*.	and *j* ≠ *k*.
	*g*_*i*_ − *g*_*oi*_ > *x*_*i*_,	*g*_*i*_ − *g*_*oi*_ + *ρf* > *x*_*i*_,	*g*_*i*_ − *g*_*oi*_ > *x*_*i*_,
	and *g*_*j*_ < *x*_*j*_,	and *g*_*j*_ + *ρf* < *x*_*j*_,	and *g*_*j*_ < *x*_*j*_,
	when *k* is a terminal	when *k* is a terminal	when *k* is a terminal
	and *j* = *k*.	and *j* = *k*.	and *j* = *k*.
	where $j\in U_{k}\cup \{k\}$, *j* is the defector group and *i* ∈ *U*_*j*_.		
${mix}_{D_{k}}$	$g_{i_{1}}-g_{oi_{1}}>x_{i_{1}}$,	$g_{i_{1}}-g_{oi_{1}}+\rho f>x_{i_{1}}$,	$g_{i_{1}}-g_{oi_{1}}>x_{i_{1}}$,
	$g_{i_{2}}>x_{i_{2}}$,	$g_{i_{2}}+\rho f>x_{i_{2}}$,	$g_{i_{2}}>x_{i_{2}}$,
	$g_{i_{3}}-(1-c_{oi_{3}})g_{oi_{3}}<x_{i_{3}}$,	$g_{i_{3}}-(1-c_{oi_{3}})g_{oi_{3}}+\rho f<x_{i_{3}}$,	$g_{i_{3}}-(1-c_{oi_{3}})g_{oi_{3}}<x_{i_{3}}$,
	and $g_{i_{4}}<x_{i_{4}}$	and $g_{i_{4}}+\rho f<x_{i_{4}}$	and $g_{i_{4}}<x_{i_{4}}$
	here $i_{1}\in U_{k}\cup \{k\}\cup r_{k}$, *i*_2_ ∈ *s*_*k*_, *i*_3_ ∈ *w*_*k*_ and *i*_4_ ∈ *z*_*k*_

**Table 8.** The payoff matrix in defector sanction system for premier.

cases	all being cooperator except the premier	a defector after the premier
Premier cooperator	*b*_*p*_ − *x*_*p*_	*b*_*p*_ − *x*_*p*_ − *g*_*op*_
Premier defector	*b*_*p*_ − *g*_*p*_ − *ρf*	*b*_*p*_ − *g*_*p*_ − *ρf*

**Table 9.** The payoff matrix in the defector sanction system for *k* ≠ *p*.

cases	all being cooperator in **O**_ *k*_	a defector in *U*_*k*_	a defector in *D*_*k*_
*k* cooperator	*b*_*k*_ − *x*_*k*_ − *g*_*nk*_	−*g*_*k*_ − *g*_*nk*_	*b*_*k*_ − *x*_*k*_ − *g*_*ok*_ − *g*_*nk*_
*k* defector	*b*_*k*_ − *g*_*k*_ − *g*_*nk*_ − *ρf*	−*g*_*k*_ − *g*_*nk*_	*b*_*k*_ − *g*_*k*_ − *g*_*nk*_ − *ρf*

**Table 10.** The payoff matrix in the defector sanction system for *n* in terminal.

cases	all being cooperator in **O**_*n*_	A defector in *U*_*n*_
*n* cooperator	*b*_*n*_ − *x*_*n*_ − *g*_*nn*_	−*g*_*n*_ − *g*_*nn*_
*n* defector	*b*_*n*_ − *g*_*n*_ − *g*_*nn*_ − *ρf*	−*g*_*n*_ − *g*_*nn*_

Any player *k*’s payoff when *k* is neither premier nor a terminal, and *k* is a cooperator, $\Pi_{ck}$, is *c*_*ok*_(*b*_*k*_ − *x*_*k*_) − *d*_*uk*_*g*_*k*_ + *d*_*dk*_(*b*_*k*_ − *x*_*k*_ − *g*_*ok*_) − *g*_*nk*_. When *k* is a defector the payoff, $\Pi_{dk}$, is *c*_*ok*_(*b*_*k*_ − *g*_*k*_ − *ρf*) − *d*_*uk*_*g*_*k*_ + *d*_*dk*_(*b*_*k*_ − *g*_*k*_ − *ρf*) − *g*_*nk*_. Therefore, the replicator equation is,

$$\frac{dk_{c}}{dt}=k_{c}(1-k_{c})(\Pi_{ck}-\Pi_{dk})=k_{c}(1-k_{c})\{c_{ok}g_{ok}+(1-d_{uk})(g_{k}+\rho f-x_{k}-g_{ok})\}.(B\;2)$$

Any player *n*’s payoff when *n* is a terminal, and *n* is a cooperator, $\Pi_{cn}$, is *c*_*on*_(*b*_*n*_ − *x*_*n*_) − *d*_*un*_*g*_*n*_ − *g*_*nn*_. When *n* is a defector the payoff, $\Pi_{dn}$, is, *c*_*on*_(*b*_*n*_ − *g*_*n*_ − *ρf*) − *d*_*un*_*g*_*n*_ − *g*_*nn*_. Therefore, the replicator equation is,

$$\frac{dn_{c}}{dt}=n_{c}(1-n_{c})(\Pi_{cn}-\Pi_{dn})=n_{c}(1-n_{c})\{c_{on}(g_{n}+\rho f-x_{n})\}.(B\;3)$$

Here, the benefit *b*_*k*_ given by a cooperator of the upstream as well as the term *g*_*nk*_ which represents the relation of *k*s dynamics with *N*_*k*_ are both canceled in the replicator equation. Therefore, they do not have any effect on the dynamics.

On the basis of equations (B 1)–(B 3), we make the time differential equations for $O_{k}\cup \{k\}$. Then, we calculate the Jacobian matrix from the differential equations for analysing the local stability of the equilibria based on $O_{k}\cup \{k\}$.

The Jacobian matrix for *premierD* is *J*(*premierD*). If *ξ* is a main diagonal component of *J*(*premierD*) then,

$$\xi =\left\{ \begin{array}{cc} g_{\;p}-x_{\;p}-(1-c_{op})g_{op}+\rho f,\; & \mathrm{if}\;p\;\mathrm{is}\;\mathrm{the}\;\mathrm{premier}\;\mathrm{group}\;\\ 0,\; & \mathrm{otherwise}.\; \end{array}\right.$$

When *g*_*p*_ − (1 − *c*_*op*_)*g*_*op*_ + *ρf* < *x*_*p*_, *PremierD* is locally stable.

In the defector sanction system, the Jacobian matrix for *allc*_*k*_ is *J*(*allc*_*k*_). If *ξ* is a main diagonal component of *J*(*allc*_*k*_) then

$$\xi =\left\{ \begin{array}{cc} x_{i}-g_{i}-\rho f,\; & \mathrm{if}\;is\;\mathrm{are}\;\mathrm{the}\;\mathrm{groups}\;\mathrm{in}\;O_{k}\;\\ 0,\; & \mathrm{otherwise}.\; \end{array}\right.$$

When *x*_*i*_ < *g*_*i*_ + *ρf*, for all *i*s, *allc*_*k*_ is stable.

The Jacobian matrix for ${mix}_{U_{k}\cup \{k\}}$ is $J({mix}_{U_{k}\cup \{k\}})$. If *ξ* is a main diagonal component of $J({mix}_{U_{k}\cup \{k\}})$ and *k* is not a terminal, or *k* is a terminal however, *k* is not the defector group, then

$$\xi =\left\{ \begin{array}{cc} x_{i}-g_{i}+g_{oi}-\rho f,\; & \mathrm{if}\;is\;\mathrm{are}\;\mathrm{the}\;\mathrm{groups}\;\mathrm{in}\;O_{k}\;\mathrm{that}\;\mathrm{are}\;\mathrm{full}\;\mathrm{cooperators}\;\\ g_{\;j}-x_{\;j}-(1-c_{oj})g_{oj}+\rho f,\; & \mathrm{if}\;js\;\mathrm{are}\;\mathrm{the}\;\mathrm{group}\;\mathrm{in}\;O_{k}\;\mathrm{that}\;\mathrm{are}\;\mathrm{full}\;\mathrm{defector}\;\\ 0,\; & \mathrm{otherwise}.\; \end{array}\right.$$

When *x*_*i*_ < *g*_*i*_ − *g*_*oi*_ + *ρf* for all *i*s, and *g*_*j*_ − (1 − *c*_*oj*_)*g*_*oj*_ + *ρf* < *x*_*j*_, then here the ${mix}_{U_{k}\cup \{k\}}$ is stable.

If *ξ* is a main diagonal component of $J({mix}_{U_{k}\cup \{k\}})$ and *k* is a terminal and *k* is the defector group, then

$$\xi =\left\{ \begin{array}{cc} x_{i}-g_{i}+g_{oi}-\rho f,\; & \mathrm{if}\;is\;\mathrm{are}\;\mathrm{the}\;\mathrm{groups}\;\mathrm{in}\;O_{k}\;\mathrm{that}\;\mathrm{are}\;\mathrm{full}\;\mathrm{cooperators}\;\\ g_{\;j}-x_{\;j}+\rho f,\; & \mathrm{if}\;j\;\mathrm{is}\;\mathrm{the}\;\mathrm{group}\;k\;\mathrm{that}\;\mathrm{is}\;\mathrm{full}\;\mathrm{defector}.\; \end{array}\right.$$

The Jacobian matrix for ${mix}_{D_{k}}$ is $J({mix}_{D_{k}})$. If *ξ* is a main diagonal component of $J({mix}_{D_{k}})$ then

$$\xi =\left\{ \begin{array}{cc} x_{i_{1}}-g_{i_{1}}+g_{oi_{1}}-\rho f,\; & \mathrm{if}\;i_{1}s\;\mathrm{are}\;\mathrm{the}\;\mathrm{groups}\;\mathrm{in}\;U_{k}\cup \{k\}\cup r_{k}\;\\ x_{i_{2}}-g_{i_{2}}-\rho f,\; & \mathrm{if}\;i_{2}s\;\mathrm{are}\;\mathrm{the}\;\mathrm{groups}\;\mathrm{in}\;s_{k}\;\\ g_{i_{3}}-x_{i_{3}}-(1-c_{oi_{3}})g_{oi_{3}}+\rho f,\; & \mathrm{if}\;i_{3}s\;\mathrm{are}\;\mathrm{the}\;\mathrm{groups}\;\mathrm{in}\;w_{k}\;\\ g_{i_{4}}-x_{i_{4}}+\rho f,\; & \mathrm{if}\;i_{4}s\;\mathrm{are}\;\mathrm{the}\;\mathrm{groups}\;\mathrm{in}\;z_{k}\;\\ 0,\; & \mathrm{otherwise}.\; \end{array}\right.$$

When $g_{i_{1}}-g_{oi_{1}}+\rho f>x_{i_{1}}$ and ($g_{i_{3}}-(1-c_{oi_{3}})g_{oi_{3}}+\rho f<x_{i_{3}}$ or $g_{i_{4}}+\rho f<x_{i_{4}}$) and/or $g_{i_{2}}+\rho f>x_{i_{2}}$, then the ${mix}_{D_{k}}$ is stable. Table 7 summarizes the local stability condition of four equilibrium points in the defector sanction system.

## Appendix C. The premier sanction system

The payoff matrices of the premier sanction system are shown in tables 11–13. The payoffs are the same as the baseline except while there is a defection in downstream the player in the premier group is subjected to the sanction *f*. Premier’s payoff in the premier sanction system when he is a cooperator, $\Pi_{cp}$, is *b*_*p*_ − *x*_*p*_ − (1 − *c*_*op*_)(*g*_*op*_ + *f*). Premier’s payoff in the premier sanction system when he is a defector, $\Pi_{dp}$, is *b*_*p*_ − *g*_*p*_ − *f*. Therefore, the replicator equation is,

$$\frac{dp_{c}}{dt}=p_{c}(1-p_{c})(\Pi_{cp}-\Pi_{dp})=p_{c}(1-p_{c})(c_{op}(g_{op}+f)+g_{\;p}-g_{op}-x_{\;p}).(C\;1)$$

Any player *k*’s payoff when *k* is neither premier nor a terminal, and *k* is a cooperator, $\Pi_{ck}$, is *c*_*ok*_(*b*_*k*_ − *x*_*k*_) − *d*_*uk*_*g*_*k*_ + *d*_*dk*_(*b*_*k*_ − *x*_*k*_ − *g*_*ok*_) − *g*_*nk*_. When *k* is a defector the payoff $\Pi_{dk}$, is *c*_*ok*_(*b*_*k*_ − *g*_*k*_) − *d*_*uk*_*g*_*k*_ + *d*_*dk*_(*b*_*k*_ − *g*_*k*_) − *g*_*nk*_. Therefore, the replicator equation is,

$$\frac{dk_{c}}{dt}=k_{c}(1-k_{c})(\Pi_{ck}-\Pi_{dk})=k_{c}(1-k_{c})\{c_{ok}g_{ok}+(1-d_{uk})(g_{k}-x_{k}-g_{ok})\}.(C\;2)$$

**Table 11.** The payoff matrix in premier sanction system for premier.

cases	all being cooperator except the premier	a defector after the premier
Premier cooperator	*b*_*p*_ − *x*_*p*_	*b*_*p*_ − *x*_*p*_ − *g*_*op*_ − *f*
Premier defector	*b*_*p*_ − *g*_*p*_ − *f*	*b*_*p*_ − *g*_*p*_ − *f*

**Table 12.** The payoff matrix in the premier sanction system for *k* ≠ *p*.

cases	all being cooperator in **O**_ *k*_	a defector in *U*_*k*_	a defector in *D*_*k*_
*k* cooperator	*b*_*k*_ − *x*_*k*_ − *g*_*nk*_	−*g*_*k*_ − *g*_*nk*_	*b*_*k*_ − *x*_*k*_ − *g*_*ok*_ − *g*_*nk*_
*k* defector	*b*_*k*_ − *g*_*k*_ − *g*_*nk*_	−*g*_*k*_ − *g*_*nk*_	*b*_*k*_ − *g*_*k*_ − *g*_*nk*_

**Table 13.** The payoff matrix in the premier sanction system for *n* in terminal.

cases	all being cooperator in **O**_*n*_	a defector in *U*_*n*_
*n* cooperator	*b*_*n*_ − *x*_*n*_ − *g*_*nn*_	−*g*_*n*_ − *g*_*nn*_
*n* defector	*b*_*n*_ − *g*_*n*_ − *g*_*nn*_	−*g*_*n*_ − *g*_*nn*_

Any player *n*’s payoff when *n* is a terminal, and *n* is a cooperator, $\Pi_{cn}$, is *c*_*on*_(*b*_*n*_ − *x*_*n*_) − *d*_*un*_*g*_*n*_ − *g*_*nn*_. When *n* is a defector the payoff, $\Pi_{dn}$, is *c*_*on*_(*b*_*n*_ − *g*_*n*_) − *d*_*un*_*g*_*n*_ − *g*_*nn*_. Therefore, the replicator equation is,

$$\frac{dn_{c}}{dt}=n_{c}(1-n_{c})(\Pi_{cn}-\Pi_{dn})=n_{c}(1-n_{c})\{c_{on}(g_{n}-x_{n})\}.(C\;3)$$

Here, the benefit *b*_*k*_ given by a cooperator of the upstream as well as the term *g*_*nk*_ which represents the relation of *k*s dynamics with *N*_*k*_ are both cancelled in the replicator equation. Therefore, they do not have any effect on the dynamics.

On the basis of equations (C 1)–(C 3), we make the time differential equations for $O_{k}\cup \{k\}$. Then, we calculate Jacobian matrix from the differential equations for analysing the local stability of the equilibria based on $O_{k}\cup \{k\}$. The Jacobian matrix for *premierD* is *J*(*premierD*). If *ξ* is a main diagonal component of *J*(*premierD*) then,

$$\xi =\left\{ \begin{array}{cc} g_{\;p}-x_{\;p}-(1-c_{op})g_{op}+c_{op}f,\; & \mathrm{if}\;p\;\mathrm{is}\;\mathrm{the}\;\mathrm{premier}\;\mathrm{group}\;\\ 0,\; & \mathrm{otherwise}.\; \end{array}\right.$$

When *g*_*p*_ − (1 − *c*_*op*_)*g*_*op*_ + *c*_*op*_*f* < *x*_*p*_, *PremierD* is locally stable.

In the premier sanction system the Jacobian matrix for *allc*_*k*_ is *J*(*allc*_*k*_). If *ξ* is a main diagonal component of *J*(*allc*_*k*_) then

$$\xi =\left\{ \begin{array}{cc} x_{\;p}-g_{\;p}-f,\; & \mathrm{if}\;p\;\mathrm{is}\;\mathrm{the}\;\mathrm{premier}\;\mathrm{group}\;\\ x_{i}-g_{i},\; & \mathrm{if}\;is\;\mathrm{are}\;\mathrm{the}\;\mathrm{groups}\;\mathrm{in}\;O_{k}\;\\ 0,\; & \mathrm{otherwise}.\; \end{array}\right.$$

As the model has the social dilemma situation, *x*_*i*_ > *g*_*i*_. Then, *allc*_*k*_ is not locally stable.

The Jacobian matrix for ${mix}_{U_{k}\cup \{k\}}$ is $J({mix}_{U_{k}\cup \{k\}})$. If *ξ* is a main diagonal component of $J({mix}_{U_{k}\cup \{k\}})$ and *k* is not a terminal, or *k* is a terminal however, *k* is not the defector group, then

$$\xi =\left\{ \begin{array}{cc} x_{i}-g_{i}+g_{oi},\; & \mathrm{if}\;is\;\mathrm{are}\;\mathrm{the}\;\mathrm{groups}\;\mathrm{in}\;O_{k}\;\mathrm{that}\;\mathrm{are}\;\mathrm{full}\;\mathrm{cooperators}\;\\ g_{\;j}-x_{\;j}-(1-c_{oj})g_{oj},\; & \mathrm{if}\;j\;\mathrm{is}\;\mathrm{the}\;\mathrm{group}\;\mathrm{in}\;O_{k}\;\mathrm{that}\;\mathrm{is}\;\mathrm{full}\;\mathrm{defector}\;\\ 0,\; & \mathrm{otherwise}.\; \end{array}\right.$$

If *ξ* is a main diagonal component of $J({mix}_{U_{k}\cup \{k\}})$ and *k* is a terminal and *k* is the defector group, then

$$\xi =\xi =\{\begin{array}{cc} x_{i}-g_{i}+g_{oi},\; & \mathrm{if}\;is\;\mathrm{are}\;\mathrm{the}\;\mathrm{groups}\;\mathrm{in}\;O_{k}\;\mathrm{that}\;\mathrm{are}\;\mathrm{full}\;\mathrm{cooperators}\;\\ g_{j}-x_{j},\; & \mathrm{if}\;j\;\mathrm{is}\;\mathrm{the}\;\mathrm{group}\;k\;\mathrm{that}\;\mathrm{is}\;\mathrm{full}\;\mathrm{defector}\; \end{array}$$

As the model has the social dilemma situation, *x*_*i*_ > *g*_*i*_ − *g*_*oi*_. Therefore, the ${mix}_{U_{k}\cup \{k\}}$ is not stable.

The Jacobian matrix for ${mix}_{D_{k}}$ is $J({mix}_{D_{k}})$. If *ξ* is a main diagonal component of $J({mix}_{D_{k}})$ then

$$\xi =\xi =\left\{ \begin{array}{cc} x_{i_{1}}-g_{i_{1}}+g_{oi_{1}},\;\; & \mathrm{if}\;i_{1}s\;\mathrm{are}\;\mathrm{the}\;\mathrm{groups}\;\mathrm{in}\;U_{k}\cup \{k\}\cup r_{k}\;\\ x_{i_{2}}-g_{i_{2}},\;\; & \mathrm{if}\;i_{2}s\;\mathrm{are}\;\mathrm{the}\;\mathrm{groups}\;\mathrm{in}\;s_{k}\;\\ g_{i_{3}}-x_{i_{3}}-(1-c_{oi_{3}})g_{oi_{3}},\;\; & \mathrm{if}\;i_{3}s\;\mathrm{are}\;\mathrm{the}\;\mathrm{groups}\;\mathrm{in}\;w_{k}\;\\ g_{i_{4}}-x_{i_{4}},\;\; & \mathrm{if}\;i_{4}s\;\mathrm{are}\;\mathrm{the}\;\mathrm{groups}\;\mathrm{in}\;z_{k}\;\\ 0,\;\; & \mathrm{otherwise}.\; \end{array}\right.$$

This equilibrium is always unstable as the model has the social dilemma situation, $x_{i_{1}}>g_{i_{1}}-g_{oi_{1}}$, because for $J({mix}_{D_{k}})$, $U_{k}\cup \{k\}$ is never an empty set. Table 7 summarizes the local stability condition of four equilibrium points in the premier sanction system.

## Appendix D. Impact of added levels and branches in a network

**D.1. The effect of the added levels on the evolution of cooperation**

In order to see the effects of the added levels of branching downstream on the evolution of cooperation, we focus on the stability conditions of the equilibria (tables 5 and 7). We find that, in the defector sanction system when a group *i* is not a terminal, whether it will be a full defector group in the equilibria depends on the condition *g*_*i*_ − (1 − *c*_*oi*_)*g*_*oi*_ + *ρf* < *x*_*i*_. It stands for the premier group in the stability condition for *premierD* equilibrium as well as for the other groups in the stability condition for the mixed equilibria. When we add another level of branching downstream of the same network, in the mentioned inequality, *g*_*i*_ and *x*_*i*_ do not change, however, *c*_*oi*_ and *g*_*oi*_ would change in group *i*. When levels are added downstream in the same network, *c*_*oi*_ decreases or stays the same by definition. So, we need to focus on *g*_*oi*_ to find out how it changes and that change will influence the local stability condition of *premierD* and the mixed equilibria.

We take a two regular once branched tree network, where the premier node is given with *p*, and the two other nodes downstream are *k*_1_ and *k*_2_ (figure 8*a*). The potential loss for the premier in the 1-level is (*g*_*op*_)_1_. Then we add one more level of branching, to this exact network’s downstream, with the branches of *k*_1_ being *k*_11_, and *k*_12_, *k*_2_ being *k*_21_, and *k*_22_ as shown in figure 2. The potential loss for the premier in this network with two levels is (*g*_*op*_)_2_. Now we take the difference between this two potential losses.

$$\begin{array}{rl} {\left(g_{op}\right)}_{2}-{\left(g_{op}\right)}_{1} & =(1-k_{1_{c}})(g_{k_{1}})+k_{1_{c}}{\left(g_{ok_{1}}\right)}_{2}+(1-k_{2_{c}})(g_{k_{2}})+k_{2_{c}}{\left(g_{ok_{2}}\right)}_{2}-[(1-k_{1_{c}})(g_{k_{1}})+(1-k_{2_{c}})(g_{k_{2}})]\\ & =k_{1_{c}}{\left(g_{ok_{1}}\right)}_{2}+k_{2_{c}}{\left(g_{ok_{2}}\right)}_{2}\geq 0. \end{array}$$

This means that the difference between (*g*_*op*_)_2_ and (*g*_*op*_)_1_ is always going to be non-negative as the potential losses do not exist in the terminal level groups. This is the idea behind the proof in the general case.

To prove this inequality in the general case, we take an arbitrary node in a tree graph network indexed $k_{\;j_{0}}$ which is followed by *n* number of levels of branching downstream, meaning the groups in the *n*th level are the terminals (figure 9). Keeping every parameter for every existing group the same, we add another level downstream of the same network, thus it becomes *n* levels network. The potential loss to $k_{\;j_{0}}$ in the *n* + 1 levels network is ${\left(g_{ok_{\;j_{0}}}\right)}_{\left(n+1\right)}$, and to $k_{\;j_{0}}$ in the *n* levels network is ${\left(g_{ok_{\;j_{0}}}\right)}_{\left(n\right)}$. We will prove that ${\left(g_{ok_{\;j_{0}}}\right)}_{\left(n+1\right)}-{\left(g_{ok_{\;j_{0}}}\right)}_{\left(n\right)}\geq 0$ in the following. Through this proof, we find that the potential losses to each level of groups are greater than or equal to what they were before when a level is added to the same network. In other words, for a group *i* in any level other than the terminal, (*g*_*oi*_)_(*n*+1)_ ≥ (*g*_*oi*_)_(*n*)_. If a level is added in a *n* levels network, (*c*_*oi*_)_(*n*+1)_ ≤ (*c*_*oi*_)_(*n*)_ holds according to the definition. The local stability analysis where group *i* is not terminal indicates that the cooperation is less evolved in group *i* if (1 − *c*_*oi*_) and *g*_*oi*_ is higher (tables 5 and 7). Therefore, adding a level downstream in the same network hinders the evolution of cooperation.

Proof. Figure 9 shows that the downstream branches of $k_{\;j_{0}}$ includes from $k_{(j_{0})1}$ to $k_{(j_{0})m_{1}}$, which set is indexed in the summation with $k_{\;j_{1}}$. Therefore, the first level of branching is indexed with *j*_1_. This indexing goes on until the (*n* + 1)th level, where the $k_{(j_{n})1}$ to $k_{(j_{n})m_{n+1}}$ are indexed with $k_{\;j_{n+1}}$. Here, $m_{1},\ldots ,\;m_{n+1}\in N$ the set of natural numbers, which count the number of branches coming out of $k_{\;j_{0}},\;k_{\;j_{1}},\ldots ,\;k_{\;j_{n}}$, respectively. The proof is as follows:

$$\begin{array}{rl} & {\left(g_{ok_{\;j_{0}}}\right)}_{\left(n+1\right)}-{\left(g_{ok_{\;j_{0}}}\right)}_{\left(n\right)}\\ & \;=[(1-k_{(j_{0})1})g_{k_{(j_{0})1}}+k_{(j_{0})1}{\left(g_{ok_{(j_{0})1}}\right)}_{\left(n+1\right)}+\cdot \;\cdot \;\cdot +(1-k_{(j_{0})m_{1}})g_{k_{(j_{0})m_{1}}}+k_{(j_{0})m_{1}}{\left(g_{ok_{(j_{0})m_{1}}}\right)}_{\left(n+1\right)}]\\ & \;-[(1-k_{(j_{0})1})g_{k_{(j_{0})1}}+k_{(j_{0})1}{\left(g_{ok_{(j_{0})1}}\right)}_{\left(n\right)}+\cdot \;\cdot \;\cdot +(1-k_{(j_{0})m_{1}})g_{k_{(j_{0})m_{1}}}+k_{(j_{0})m_{1}}{\left(g_{ok_{(j_{0})m_{1}}}\right)}_{\left(n\right)}]\\ & \;=\sum_{\;j_{1}=(j_{0})1}^{(j_{0})m_{1}}k_{\;j_{1_{c}}}\{{\left(g_{ok_{\;j_{1}}}\right)}_{\left(n+1\right)}-{\left(g_{ok_{\;j_{1}}}\right)}_{\left(n\right)}\}\\ & \;=\sum_{\;j_{1}=(j_{0})1}^{(j_{0})m_{1}}k_{\;j_{1_{c}}}\left\{\sum_{\;j_{2}=(j_{1})1}^{(j_{1})m_{2}}k_{\;j_{2_{c}}}\{{\left(g_{ok_{\;j_{2}}}\right)}_{\left(n+1\right)}-{\left(g_{ok_{\;j_{2}}}\right)}_{\left(n\right)}\}\right\}\\ & \;\;\cdot \\ & \;\;\cdot \\ & \;\;\cdot \\ & =\sum_{\;j_{1}=(j_{0})1}^{(j_{0})m_{1}}k_{\;j_{1_{c}}}\left\{\sum_{\;j_{2}=(j_{1})1}^{(j_{1})m_{2}}k_{\;j_{2_{c}}}\left\{\cdot \cdot \cdot \sum_{\;j_{n-1}=(j_{n-2})1}^{(j_{n-2})m_{n-1}}k_{{\left(j_{n-1}\right)}_{c}}\{{\left(g_{ok_{\;j_{n-1}}}\right)}_{\left(n+1\right)}-{\left(g_{ok_{\;j_{n-1}}}\right)}_{\left(n\right)}\}\cdot \cdot \cdot \right\}\right\}\\ & \;=\sum_{\;j_{1}=(j_{0})1}^{(j_{0})m_{1}}k_{\;j_{1_{c}}}\{\sum_{\;j_{2}=(j_{1})1}^{(j_{1})m_{2}}k_{\;j_{2_{c}}}\{\cdot \cdot \cdot \sum_{\;j_{n-1}=(j_{n-2})1}^{(j_{n-2})m_{n-1}}k_{{\left(j_{n-1}\right)}_{c}}\{(1-k_{(\;j_{n-1})1_{c}})g_{k_{\left(j_{n-1}\right)1}}+k_{(\;j_{n-1})1_{c}}{\left(g_{ok_{\left(j_{n-1}\right)1}}\right)}_{\left(n+1\right)}+\;\cdot \cdot \cdot \\ & \;\;+(1-k_{(\;j_{n-1}){m_{n}}_{c}})g_{k_{\left(j_{n-1}\right)m_{n}}}+k_{(\;j_{n-1}){m_{n}}_{c}}{\left(g_{ok_{\left(j_{n-1}\right)m_{n}}}\right)}_{\left(n+1\right)}-\{(1-k_{(\;j_{n-1})1_{c}})g_{k_{\left(j_{n-1}\right)1}}+\;\cdot \cdot \cdot \\ & \;\;+(1-k_{(\;j_{n-1}){m_{n}}_{c}})g_{k_{\left(j_{n-1}\right)m_{n}}}\}\}\cdot \cdot \cdot \}\}\\ & \;=\sum_{\;j_{1}=(j_{0})1}^{(j_{0})m_{1}}k_{\;j_{1_{c}}}\{\sum_{\;j_{2}=(j_{1})1}^{(j_{1})m_{2}}k_{\;j_{2_{c}}}\{\cdot \cdot \cdot \sum_{\;j_{n-1}=(j_{n-2})1}^{(j_{n-2})m_{n-1}}k_{{\left(j_{n-1}\right)}_{c}}\{k_{(\;j_{n-1})1_{c}}{\left(g_{ok_{\left(j_{n-1}\right)1}}\right)}_{\left(n+1\right)}\\ & \;\;+\cdot \cdot \cdot +k_{(\;j_{n-1}){m_{n}}_{c}}{\left(g_{ok_{\left(j_{n-1}\right)m_{n}}}\right)}_{\left(n+1\right)}\}\cdot \cdot \cdot \}\}\\ & \;=\sum_{\;j_{1}=(j_{0})1}^{(j_{0})m_{1}}k_{\;j_{1_{c}}}\left\{\sum_{\;j_{2}=(j_{1})1}^{(j_{1})m_{2}}k_{\;j_{2_{c}}}\left\{\cdot \cdot \cdot \sum_{\;j_{n-1}=(j_{n-2})1}^{(j_{n-2})m_{n-1}}k_{{\left(j_{n-1}\right)}_{c}}\left\{\sum_{\;j_{n}=(j_{n-1})1}^{(j_{n-1})m_{n}}k_{\;j_{n_{c}}}g_{ok_{\;j_{n}}}\right\}\cdot \cdot \cdot \right\}\right\}\\ & \;\geq 0. \end{array}$$

The key point of this proof is to use ${\left(g_{ok_{\left(j_{n-1}\right)i}}\right)}_{\left(n\right)}=0$ where *i* is $1,\;\ldots ,\;m_{n}$, and $k_{\left(j_{n-1}\right)i}$s are groups in the *n*th level from group $k_{\;j_{0}}$, and are the terminals when the network has *n* or more number of levels.  ▪

**D.2. The effect of the added branches on the evolution of cooperation**

However, the same as the previous section cannot be said when a branch is added parallelly to a network. If we compare a one-level two-branched network (figure 10*a*) with a one-level three-branched network (figure 10*b*) from the perspective of the premier *p*, it becomes clear. Let the potential loss of the premier in the former be (*g*_*op*_)_(2)_ and the latter be (*g*_*op*_)_(3)_. The terminals of the former be *k*_1_ and *k*_2_, and in the latter *k*_3_ is added in parallel to them.

$$\begin{array}{rl} & {\left(g_{op}\right)}_{\left(3\right)}-{\left(g_{op}\right)}_{\left(2\right)}=(1-k_{1_{c}}){\left(g_{k_{1}}\right)}_{\left(3\right)}+(1-k_{2_{c}}){\left(g_{k_{2}}\right)}_{\left(3\right)}+(1-k_{3_{c}}){\left(g_{k_{3}}\right)}_{\left(3\right)}-(1-k_{1_{c}}){\left(g_{k_{1}}\right)}_{\left(2\right)}-(1-k_{2_{c}}){\left(g_{k_{2}}\right)}_{\left(2\right)}\\ & =\alpha . \end{array}$$

The value *α* can take any real value. The only real constraint here is that ${\left(g_{k_{1}}\right)}_{\left(3\right)}+{\left(g_{k_{2}}\right)}_{\left(3\right)}+$ ${\left(g_{k_{3}}\right)}_{\left(3\right)}=g_{\;p}={\left(g_{k_{1}}\right)}_{\left(2\right)}+{\left(g_{k_{2}}\right)}_{\left(2\right)}$, however, this condition is not enough to prove *α* being either positive or negative or zero. Therefore, the effect of the addition of a branch parallelly in a network is not determinable.
